# Metabolomic Analysis Demonstrates the Impacts of Polyketide Synthases PKS14 and PKS15 on the Production of Beauvericins, Bassianolide, Enniatin A, and Ferricrocin in Entomopathogen *Beauveria bassiana*

**DOI:** 10.3390/metabo13030425

**Published:** 2023-03-14

**Authors:** Wachiraporn Toopaang, Kullyanee Panyawicha, Chettida Srisuksam, Wei-Chen Hsu, Ching-Chih Lin, Morakot Tanticharoen, Yu-Liang Yang, Alongkorn Amnuaykanjanasin

**Affiliations:** 1 National Center for Genetic Engineering and Biotechnology (BIOTEC), National Science and Technology Development Agency (NSTDA), 113 Thailand Science Park, Paholyothin Rd., Khlong Nueng, Khlong Luang, Pathum Thani 12120, Thailand; 2 Molecular and Biological Agricultural Sciences, Taiwan International Graduate Program, Academia Sinica and National Chung Hsing University, Taiwan; 3 Agricultural Biotechnology Research Center, Academia Sinica, Taipei 11529, Taiwan; 4 Institute of Biotechnology, College of Bioresources and Agriculture, National Taiwan University, Taipei 10617, Taiwan; 5 Biotechnology Center in Southern Taiwan, Academia Sinica, Tainan 711010, Taiwan; 6 School of Bioresources and Technology, King Mongkut’s University of Technology Thonburi, Bangkok 10140, Thailand

**Keywords:** *Beauveria bassiana*, polyketide synthase, nonribosomal peptides, GNPS, molecular networking

## Abstract

*Beauveria bassiana* is a globally distributed entomopathogenic fungus that produces various secondary metabolites to support its pathogenesis in insects. Two polyketide synthase genes, *pks14* and *pks15*, are highly conserved in entomopathogenic fungi and are important for insect virulence. However, understanding of their mechanisms in insect pathogenicity is still limited. Here, we overexpressed these two genes in *B. bassiana* and compared the metabolite profiles of *pks14* and *pks15* overexpression strains to those of their respective knockout strains in culture and in vivo using tandem liquid chromatography-mass spectrometry (LC-MS/MS) with Global Natural Products Social Molecular Networking (GNPS). The *pks14* and *pks15* clusters exhibited crosstalk with biosynthetic clusters encoding insect-virulent metabolites, including beauvericins, bassianolide, enniatin A, and the intracellular siderophore ferricrocin under certain conditions. These secondary metabolites were upregulated in the *pks14*-overexpressing strain in culture and the *pks15*-overexpressing strain in vivo. These data suggest that *pks14* and *pks15*, their proteins or their cluster components might be directly or indirectly associated with key pathways in insect pathogenesis of *B. bassiana*, particularly those related to secondary metabolism. Information about interactions between the polyketide clusters and other biosynthetic clusters improves scientific understanding about crosstalk among biosynthetic pathways and mechanisms of pathogenesis.

## 1. Introduction

*Beauveria bassiana* is an entomopathogenic fungus with a broad host range among insect pests. This fungus has great potential to control the diamondback moth, European corn borer, corn earworm, black cutworm, cabbage worm, and cabbage looper [1]. Major *B. bassiana* secondary metabolites are polyketides, nonribosomal peptides (NRPs), and hybrid polyketide NRPs, which are widely used in medical and agricultural applications, such as insecticides, antitumor applications, antibiotics, antioxidative stress treatments, and immunosuppression [2,3,4,5]. Well-known NRPs reported to be involved in insect pathogenesis include beauvericin, bassianolide, and enniatin, which are synthesized by NRP synthetases (NRPS), e.g., beauvericin synthetase, bassianolide synthetase, and enniatin synthetase [2,3,6]. These compounds have much potential for use in the management of insects such as *Galleria mellonella*, *Salix exigua*, *Helicoverpa zea*, and *Choristoneura fumiferana* [6,7,8]. In addition, ferricrocin has been considered a virulence factor in several hosts. It is an intracellular siderophore commonly found in *Aspergillus fumigatus*, *A. nidulans*, *B. bassiana*, *Metarhizium robertsii*, and *M. grisea* [9,10,11,12,13,14]. Ferricrocin has a crucial role in conidiation, conidial germination, resistance to oxidative stress, and virulence against insects [10,12,14,15].

In addition to NRPs, polyketides such as stilbenes, spinosyn A, spinosyn, avermectins, ivermectins, and anthraquinones also play important roles in insect virulence. While they are mainly found in bacteria, insecticidal polyketides can be found in fungi as well, and encompass compounds such as dihydroxynaphthalene (DHN)-melanin, dipicolinic acid, neurosporin A, and phomalactone [16]. Noticeably, entomopathogenic fungi have an abundance of polyketide synthases (PKS); for example, there are 24, 13, and 12 PKS genes in *M. robertsii* (ARSEF 2575), *M. acridum*, and *B. bassiana*, respectively [17,18]. However, study of their functions and mechanisms in controlling insect pests is still limited. Of the 12 *B*. *bassiana* BCC 2660 PKS genes, two of these, *pks14* and *pks15*, are highly conserved in entomopathogenic fungi and play a crucial role in virulence against insects and in anti-phagocytic activity against insect hemocytes [19,20,21]. Despite these findings, there is still limited knowledge about how *B. bassiana* polyketides are involved in insect virulence. Moreover, to our knowledge, no study has demonstrated cross-relationships between polyketide clusters and other secondary metabolite clusters such as NRPs with respect to insect pathogenesis.

Mass-spectrometry-based metabolomics is a powerful analytical technique used to explore secondary metabolites in biological samples. Liquid chromatography–mass spectrometry (LC-MS) coupling of liquid chromatography and mass spectrometry are widely used in metabolomic analysis [22]. Integration of tandem mass spectrometry (MS/MS) with Global Natural Products Social Molecular Networking (GNPS) generates molecular networking by grouping similar mass spectra from MS/MS. [23]. Molecular networking not only allows the discovery of novel compounds and evaluation of differences in metabolite profiles from different sources, but also evaluates metabolite production in response to specific interactions [24,25,26].

In this study, we explored the impacts of PKS14 and PKS15 in *B. bassiana* BCC 2660 on fungal virulence by comparing mass-spectrometry-based metabolite profiles between overexpressing (OE*pks*) and knockout (∆*pks*) strains in culture media and in vivo. Molecular networking revealed the influences of PKS14 and PKS15 on the production of well-described insect-virulence NRPs, including beauvericins, enniatin A, bassianolide, and the intracellular siderophore ferricrocin. These PKSs and their metabolites may be associated with certain biosynthetic pathways of insect virulence factors, in turn helping govern the core processes of pathogenesis.

## 2. Materials and Methods

### 2.1. Fungal Strains and Culture Conditions

The knockout mutants ∆*pks14* and ∆*pks15* were previously generated in *B. bassiana* BCC 2660 and deposited in the Thailand Bioresource Research Center [19,20]. The PKS genes were disrupted by integration of the bialaphos resistance gene *bar* in the respective genes in *B. bassiana* BCC2660 using *Agrobacterium*-mediated transformation. *B*. *bassiana* wild type and all derivative strains were maintained on potato dextrose agar (PDA; Difco) at 28 °C for 7 days.

### 2.2. Generating Overexpressing Strains of pks14 and pks15 

To generate strains overexpressing *pks14* (*sGFP* + *pks14* + ::*bar*^R^) and *pks15* (*sGFP* + *pks15* + ::*bar*^R^), the green fluorescence protein gene *sGFP* fused to full-length *pks14* or *pks15* under the control of the constitutive promoter *toxA* from *Pyrenophora tritici-repentis* [27] was inserted into a vector, as previously described [28]. Briefly, the full-length *pks14* was amplified from genomic DNA from the start codon to 300 bp downstream of the stop codon with primers PKS14Eco1F (5′-GTGAATTCATGGAGCCAATCGCCATTGTCGG-3′) and PKS14Nhe7591R (5′-ATTGCTAGCGTCTGTCGAGCCGCGTCAGTG-3′). *Eco*RI and *Nhe*I sites in the primers are underlined, respectively. The *pks14* fragment was then inserted into the pToxA vector [29] at *Eco*RI and *Avr*II (compatible with *Nhe*I) to generate the pTxA-sGFP-PKS14 vector. The full-length *pks15* was amplified from genomic DNA from the start codon to 314 bp downstream of the stop codon with the primers PKSIII-start-Mfe: 5′-GGGCAATTGATGCTCATCGACAAAATGGAGACG-3′ and PKSIII-3′-NC-BamHI: 5′-TTTGGATCCCTCCCGAGTCTACCTTGATGC-3′. The *Mfe*I and *Bam*HI sites in the primers are underlined, respectively. The *pks15* fragment was inserted into the pToxA vector [30] at *Eco*RI and *Bam*HI (compatible with *Mfe*I) to generate the pTxA-sGFP-PKS15 vector. The overexpression vectors pTxA-sGFP-PKS14 and pTxA-sGFP-PKS15 were transformed individually into *B. bassiana* strain BCC 2660 using PEG-protoplast transformation [30].

Transformants were selected on minimal medium (2% *w/v* dextrose, 0.51% *w/v* (NH_4_)_2_SO_4_ (Sigma Aldrich), 0.17% *w/v* yeast nitrogen base without amino acid (Difco), 1.8% *w/v* agar) supplemented with 200 mg·L^−1^ glufosinate ammonium (Zhejiang Yongnong Chem, Wenzhou, China) and confirmed by PCR amplification. Sequence integrity of the *sGFP-pks14* or *sGFP-pks15* fusion constructs was verified by DNA sequencing (Macrogen, Seoul, Republic of Korea. For gene expression analysis of the *pks14-*overexpressing (OE*pks14*) strains compared to the wild type, each strain was cultured in PDB at 150 rpm, 28 °C, for 3 days. Total RNA was extracted using the AmbionTM TRIzol (Thermo Fisher Scientific, Waltham, MA, USA), treated with DNase I (Thermo Fisher Scientific, Waltham, MA, USA) and generated cDNA using RevertAid Reverse Transcriptase and random hexamers (Thermo Fisher Scientific, Waltham, MA, USA). Gene expression levels were quantified using reverse transcription—quantitative polymerase chain reaction (RT-qPCR) with specific primers (pks14-F5′-CTT GAT CCT GTC AGC CGA TC-3′ and pks14-R5′-GCA TAC ACG TCT CTG ATG AG-3′). The beta-tubulin gene was used as the reference. Expression levels were calculated using the 2-∆∆Ct method [31]. 

### 2.3. Phenotypic Characterization of pks14- and pks15-Overexpressing Strains 

Transformants were verified for the integration of *sGFP-pks14* or *sGFP-pks15* fusion constructs in the genomes of overexpression strains by PCR analysis with specific primers for *sGFP* (sGFP-550F: 5′-CAG CAG AAC AC C CCC ATC GGC-3′ and sGFP-R: 5′-CTT GTA CAG CTC GTC CAT GCC GTG A-3′), *pks14* (PKS14-R: 5′-CTC AAG GTA CCA GAG TAG GCT AC-3′), *pks15* (PKS15-480R: 5′-CAA GCT TCC GGT ACG ATA GTC-3′), and the *ToxA* promoter (pToxA-F: 5′-TGG AAT CCA TGG AGG AGT TCT GTA C -3′).

Conidial yield and radial growth of the overexpression strains were determined by comparison to the wild type. For conidial yield, 100 µL of a conidial suspension of 1 × 10^7^ conidia·mL^−1^ was spread on PDA for 7 days, and conidial yield was determined using a hemocytomoter. For radial growth, 10 µL of a conidial suspension of 1 × 10^7^ conidia·mL^−1^ was dropped on PDA, and colony diameter was determined on day 12 after inoculation.

The subcellular localization of PKS15 In the *pks15*-overexpressing (oE*pks15*) strains was determined by incubating the strains in diluted PDB (5% (*v/v*) in water) on a glass slide for 48 h followed by visualization using a confocal laser scanning microscope model FV1000 (Olympus), as previously described [13].

Insect virulence was determined using fourth-instar *Spodoptera exigua* (beet armyworm, BAW) larvae. For each strain, 20 BAWs were injected with 3 µL of 1 × 10^4^ conidia·mL^−1^ (30 conidia per larva) using a specialized 33-gauge needle-syringe set (Hamilton), and cumulative insect mortalities were recorded for 7 days.

### 2.4. Metabolomic Preparation of OEpks14, OEpks15, Δpks14, and Δpks15 from Culture and In Vivo Samples

For the metabolomes from culture, 1 mL of conidia at 1 × 10^8^ conidia·mL^−1^ of OE*pks14*, OE*pks15*, Δ*pks14*, and Δ*pks15* was inoculated into 2 L PDB for the overexpression strains and 3 L PDB for the knockout strains. Cultures were shaken at 110 rpm and 28 °C for 7 days. Fungal cell extracts were prepared by sonication in methanol for 20 min, followed by overnight incubation. Extraction of culture broth was performed in ethyl acetate (volume ratio 1:1) three times. Crude extracts were obtained after condensation and lyophilization.

For in vivo metabolomes, ten fourth-instar BAW larvae were injected with 3 μL of conidial suspension of OE*pks14*, OE*pks15*, Δ*pks14*, or Δ*pks15* at 1 × 10^7^ conidia·mL^−1^. Inoculated larvae were collected 3, 5, and 7 days post-inoculation (DPI). Saline-injected larvae were used as controls. Larval extracts were prepared in methanol as described above. Crude extracts were obtained after condensation and lyophilization.

### 2.5. Metabolomic Analysis Using LC-MS and LC-MS/MS 

Crude extracts at 10 mg/mL in methanol were separated by C18 (ACQUITY UPLC BEH-C18, 130 Å, 1.7 μm, 2.1 × 100 mm) with the following gradients: 0–6 min at 5–99.5% acetone nitrile (ACN), 6–8 min at 99.5% ACN, 8–8.2 min at 99.5–5% ACN, and 8.2–10 min of 5% ACN with a flow rate 0.4 mL·min^−1^. Mass data were acquired in triplicate using UPLC-HR-ESIMS (Thermo Orbitrap Elite system) and analyzed in positive and negative-mode ion detection between *m/z* 50–1500 with 30,000 resolutions. The top five ions with the highest intensities from each full mass scan were selected for collision-induced dissociation (CID) fragmentation for tandem mass data. For CID, the isolation width was 2 Da, and the selected ions were fragmented with a normalized collision energy of 30.0 or 35.0, activation Q of 0.250, activation time of 10.0, and 15,000 resolutions.

### 2.6. Molecular Networking, Chemical Classification, and Structural Elucidation

#### 2.6.1. LC-MS/MS Data Processing

LC-MS/MS raw data were converted to mzML format using MSConvert software (Part of the ProteoWizard package; ProteoWizard Software Foundation, Palo Alto, USA) before data processing in MZmine software (version 2.53), as previously described [32]. The mass detection noise level was set to 1000 for MS1 and 100 for MS2. Chromatogram building was performed using the Automated Data Analysis Pipeline (ADAP) chromatogram builder with the following settings: minimum group size of scans, 3; group intensity threshold, 1000; minimum highest intensity, 10,000; and *m/z* tolerance, 0.001 *m/z* (or 20 ppm). Chromatographic deconvolution was set with S/N threshold, 10; minimum feature height of 50,000 (20,000 for negative-ion data); coefficient/area threshold, 50; peak duration range, 0.05–0.80 min; and retention time (RT) wavelet range, 0.03–0.15 min. The mass range for MS/MS scan pairing was set to 0.02 Da, and the RT range was set to 0.2 min. The isotopic peak grouping was an *m/z* tolerance of 0.001 *m/z* (or 20 ppm) and an RT tolerance of 0.2 min. Peak alignment was generated using the join aligner with an *m/z* tolerance of 0.001 *m/z* (or 20 ppm), an RT tolerance of 0.2 min, a weight for *m/z* of 70, and a weight for RT of 30. Gap filling was performed with an intensity tolerance of 30%, an *m/z* tolerance of 0.001 *m/z* (or 20 ppm), and an RT tolerance of 0.2 min. Finally, the processed data were exported in .mgf format for MS/MS spectral information and .csv format for a feature list of MS1 *m/z*, peak retention time, and peak area information.

#### 2.6.2. Molecular Networking

Molecular networking was generated using Global Natural Products Social Molecular Networking (GNPS; https://gnps.ucsd.edu; accessed on 23 May 2022) [33]. The preprocessed data were submitted to a feature-based molecular networking workflow (version 28.2) in GNPS [34]. The precursor-ion mass tolerance and MS/MS fragment ion tolerance were set to 0.02 Da. Edges in the network were created when a cosine score was above 0.7 with at least 6 matched fragment ions. The molecular network data were visualized by Cytoscape (version 3.8.0.). Annotated metabolites were collected, and structures were confirmed with their ion fragmentation from LC-MS/MS using ChemDraw Professional 16.

## 3. Results

### 3.1. pks14- and pks15-Overexpressing Strains Exhibit Increased Insect Virulence

To characterize phenotypes of the *pks14*- and *pks15*-overexpressing strains OE*pks14* and OE*pks15*, gene expression level, conidial yield, radial growth, insect mortality, and cellular localization were determined with respect to the wild type.

**Figure 1:**
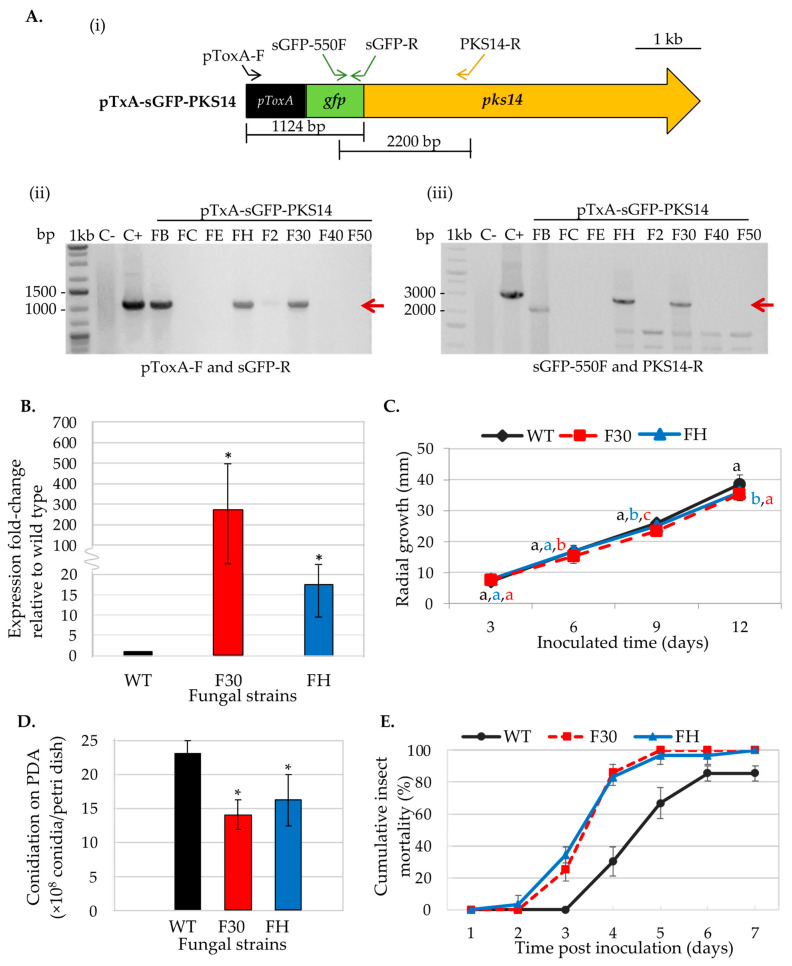
Phenotype characterization of OE*pks14* strains. (**A**) Ectopic integration of the *pks14-*overexpression cassette was verified by PCR with specific primers (**i**). OE*pks14* strains FH and F30 showed the expected bands at 1124 bp (red arrow) using primers pToxA-F and sGFP-R, specific for the *ToxA* promoter and *sGFP*, respectively (**ii**), and 2200 bp (red arrow) using primers sGFP-550F and PKS14-R, specific for the *sGFP* and *pks14*, respectively (**iii**). *B. bassiana* BCC2660 genomic DNA and the vector pTxA-sGFP-PKS14 were used as negative (C−) and positive (C+) controls, respectively. (**B**) Gene expression level, (**C**) radial growth, (**D**) conidiation, and (**E**) insect mortality were impacted by OE*pks14* strains FH and F30. The letters indicate a significant difference (ANOVA, *p* < 0.05). Asterisks indicate statistical significance (*t*-test, *p* < 0.05).

The pTxA-sGFP-PKS14 vector designed for *pks14* overexpression was transformed into the *B. bassiana* wild type. Eight transformants were obtained and examined for ectopic integration of the overexpression cassette by PCR. *B. bassiana* BCC2660 genomic DNA and the pTxA-sGFP-PKS14 vector were included as negative and positive controls, respectively. The specific primers used for this PCR are shown in Figure 1A. Two OE*pks14* strains, FH and F30, showed the expected bands at 1124 bp (using primers pToxA-F and sGFP-R specific for the *ToxA* promoter and *sGFP*, respectively) and at 2200 bp (using primers sGFP-550F and PKS14-R specific for *sGFP* and *pks14*, respectively) (Figure 1A). Expression levels of *pks14* in OE*pks14* strains F30 and FH increased by 245-fold and 18-fold, respectively, compared to the *B. bassiana* wild type (Figure 1B). In addition, these strains exhibited a slight reduction in radial growth on days 9–12 compared to that of the wild type (Figure 1C). The conidial yield of OE*pks14* strains also showed a significant reduction by approximately 1.5-fold compared to that of wild type (Figure 1D). However, the reduced conidial yield did not affect the insect virulence of OE*pks14* strains. F30 and FH virulence increased significantly by 177–187% and 18% on days 4 and days 7 after inoculation, compared to the wild type (Figure 1E). However, the GFP fluorescence was not detected in either of the OE*pks14* strains, probably due to the nature of PKS14 or unknown complexities of this s-GFP-PKS14 fusion (e.g., N- or C-terminus fusion). Therefore, OE*pks14* strain F30, which exhibited an elevated *pks14* expression level and insect virulence, was used in subsequent analyses.

**Figure 2:**
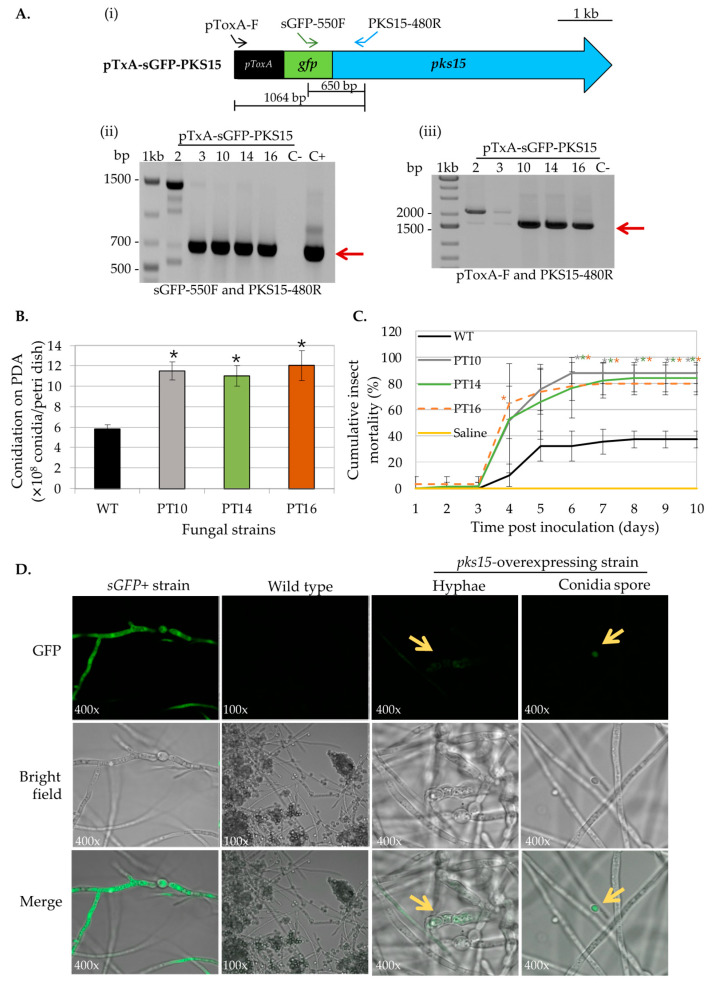
Phenotype characterization of OE*pks15* strains. (**A**) Ectopic integration of the *pks15-*overexpression cassette was verified by PCR with specific primers (**i**). The OE*pks15* strains 10, 14, and 16 showed the expected bands at 650 bp (red arrow) using primers sGFP-550F and PKS15-480R, specific for *sGFP* and *pks15*, respectively (**ii**), and 1604 bp (red arrow) using primers pToxA-F and PKS15-480R, specific for the *ToxA* promoter and *pks15*, respectively (**iii**). *B. bassiana* BCC2660 genomic DNA and the vector pTxA-sGFP-PKS15 were used as negative (C−) and positive (C+) controls, respectively. (**B**) Conidiation and (**C**) insect mortality were increased in OE*pks15* strains 10, 14, and 16. (**D**) Subcellular localization of the PKS15 in Oe*pks15* strain 16 exhibited the unique pattern of green fluorescence (GFP) in pseudohyphae-like cells and conidia (yellow arrow) compared to the negative control, *B. bassiana* wild type, and the positive control, the *B. bassiana sGFP+* strain (sGFP alone and having GFP fluorescence homogenously in the cytoplasm). Asterisks indicate statistical significance (*t*-test, *p* < 0.05).

For *pks15* overexpression, five transformants were obtained from the fungal transformation. Transformants 2, 3, 10, 14, and 16 were randomly selected for detection of ectopic integration of the overexpression cassette by PCR. The specific primers targeting the pTxA-SGFP-PKS15 vector are shown in Figure 2A. Three transformants, 10, 14, and 16, showed the expected bands at 650 bp (using primers sGFP-550F and PKS15-480R specific for *sGFP* and *pks15*, respectively) and at 1604 bp (using primers pToxA-F and PKS15-480R specific for the *ToxA* promoter and *pks15*, respectively) (Figure 2A). In addition, these three strains also had two-fold increases in conidial yield compared to wild type (Figure 2B), and their radial growths were not different (data not shown). Moreover, OE*pks15* strains 10, 14, and 16 exhibited increased insect virulence by 150%, 134%, and 128%, respectively, on day 7 compared to the wild type (Figure 2C). 

To determine the location of PKS15 in the fungal cell, cellular localization of the sGFP-PKS15 fusion protein was investigated by assessing GFP fluorescence compared to wild type and *sGFP+* (expressing sGFP alone) strains. As expected, the *B. bassiana sGFP+* strain showed uniform GFP fluorescence in the cytoplasm of both hyphae and conidia. By contrast, OE*pks15* strain 16 had a unique pattern of green fluorescence in pseudohyphae-like cells and conidia (Figure 2D). Therefore, this strain, with its evelated conidial yield and insect virulence and visible expression of the s-GFP-PKS15 fusion protein, was selected for use in subsequent analyses.

### 3.2. pks14 and pks15 Strains Differentially Express a Number of Metabolites, Including Insect Virulence Factors

Metabolite profiles of *pks14* and *pks15* overexpression and knockout strains were explored by LC-MS and LC-MS/MS. For culture medium investigation, 1 × 10^8^ conidia of each strain were inoculated in potato dextrose broth (PDB) and mass spectrometry data were collected at 3 days post-inoculation (DPI). For in vivo analyses, a suspension of 1 × 10^7^ conidia·mL^−1^ per strain was injected into beet armyworm larvae (BAW). Saline-injected BAWs were used as controls. Mass spectrometry data were collected at early-stage infection (3 DPI) when live insect larvae were being colonized by the fungus, at mid-stage infection (5 DPI) from dead larvae, and at late-stage infection (7 DPI) from cadavers covered with fungal hyphae.

To annotate fungal metabolites from these complex samples, LC-MS/MS data of crude extracts were processed by using MZmine, and molecular networking was generated on GNPS from positive and negative-ion data. Similar MS/MS spectra were grouped into the same molecular network with the precursor-ion mass tolerance of 0.02 Da, MS/MS fragment ion tolerance of 0.02 Da, a cosine score > 0.7, and peak matching > 6 peaks. The sources for each node were visualized by different colors: metabolites produced in overexpression strains in red, those from knockout strains in blue, and those from saline-injected BAWs in gray. Chromatographic peak-area proportions are represented by node sizes and pie chart distributions. Based on compound identification matching to the spectral library and MS/MS annotation, molecular families (MFs) and singletons from culture and in vivo were identified for various classes of metabolites. In the positive-ion data, 2038 nodes and 1117 MFs were obtained from cultures of *pks14* strains, whereas 2874 nodes and 1620 MFs were found in vivo. In addition, 241 nodes and 84 MFs were obtained from cultures of *pks15* strains, and 6801 nodes and 2509 MFs were found in vivo. 

**Figure 3:**
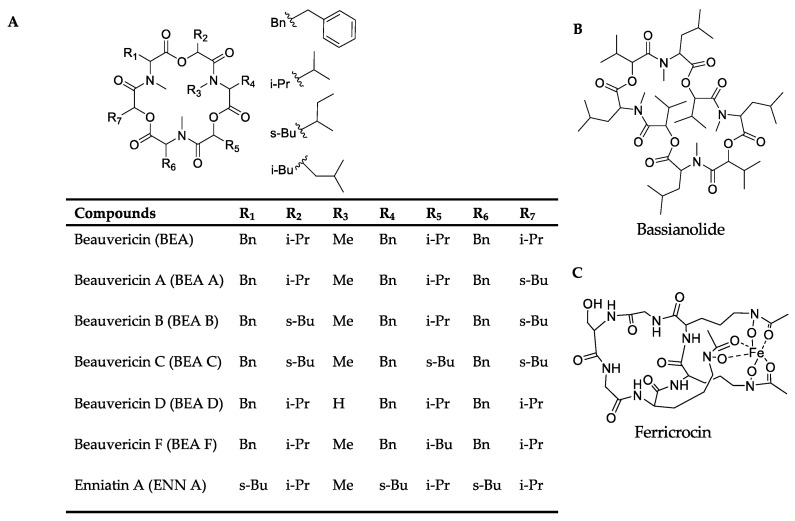
Chemical structures of (**A**) the beauvericins analyzed in this study (BEAs), enniatin A (ENN A), (**B**) bassianolide (BAS), and (**C**) ferricrocin (FER).

Among the positive-ion data, mass spectrometry data of culture samples revealed classified compounds such as aryl phosphotriester, lactone, and terpenoid exclusively from *pks14* strains, and purine nucleosides from *pks15* strains (Figures S1 and S3). For the in vivo mass spectrometry data, chemical compounds such as glycerophosphocholines, glycosides, phenolic acid, and phenylpropanes were observed exclusively for the *pks14* strains (Figure S2); and pigment was observed for the *pks15* strains (Figure S4). Moreover, compounds found in both culture and in vivo for *pks14* and *pks15* strains were amino acids, depsipeptides, dipeptides, fatty acids, fatty amides, flavins, glycerophospholipids, monoacylglycerols, pantothenic acid, and intracellular siderophores; and organophosphate was detected for the *pks14* strains (Figures S1–S4). Intriguingly, major metabolites in the depsipeptide group related to fungal virulence were identified as beauvericins (BEAs), bassianolide (BAS), enniatin A (ENN A), and ferricrocin (FER). They were detected from both the *pks14* and *pks15* strains in positive-ion data but not negative-ion data (data not shown). The major insecticides identified in this study are shown in Figure 3.

### 3.3. Insect Virulence Factors Were Found Mainly for OEpks14 in Culture 

**Figure 4:**
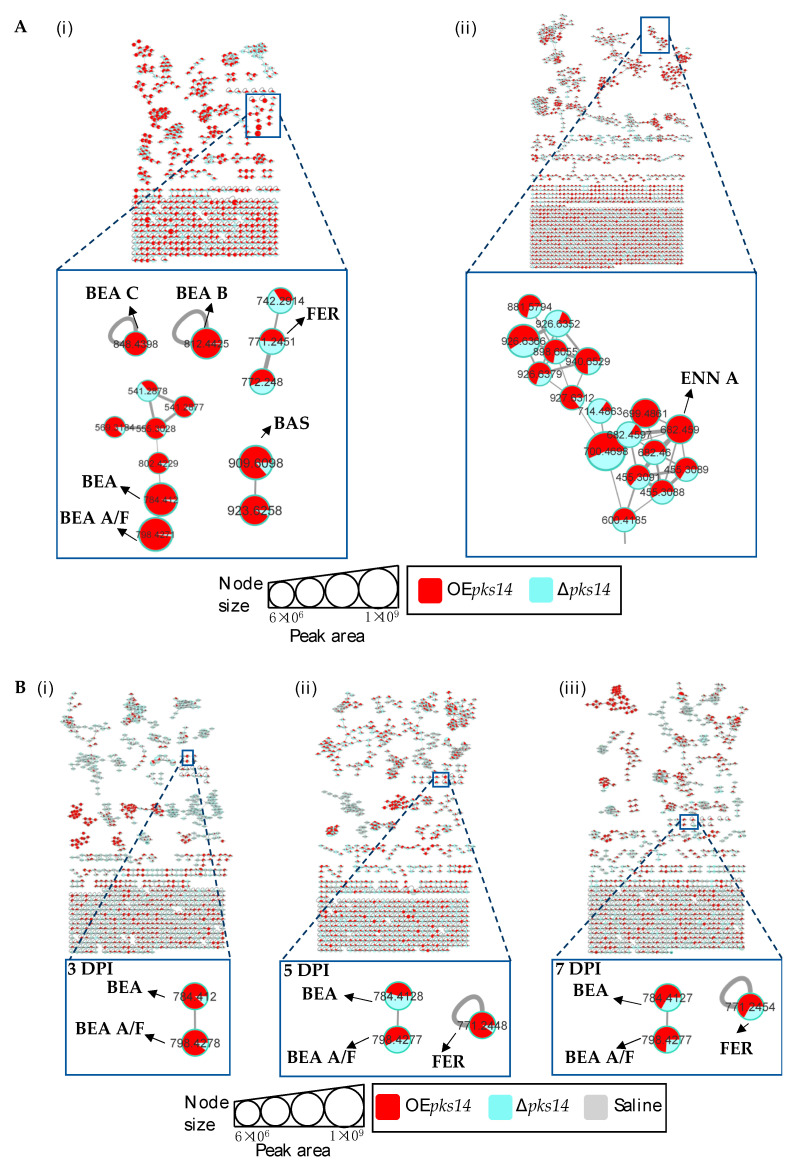
(**A**) Molecular networking of classified metabolites from OE*pks14* (red) and Δ*pks14* (light blue) strains in culture identified beauvericin (BEA), beauvericin A/F (BEA A/F), beauvericin B (BEA B), beauvericin C (BEA C), bassianolide (BAS), and ferricrocin (FER) in cells (**i**) and enniatin A (ENN A) in culture broth (**ii**). (**B**) Molecular networking of classified compounds from OE*pks14* (red) and Δ*pks14* (light blue) strains in vivo at early-stage infection (3 DPI) for live larvae (**i**), mid-stage infection (5 DPI) for dead larvae (**ii**), and late-stage infection (7 DPI) for cadavers covered with fungal hyphae (**iii**) identified beauvericin (BEA), beauvericin A/F (BEA A/F), and ferricrocin (FER). Saline-injected BAWs were used as controls (gray). Node sizes represent the sums of chromatographic peak areas, and pie charts indicate chromatographic peak-area proportions for the detected insect virulence factors.

Positive ion data of the OE*pks14* and ∆*pks14* strains in culture media were compared to assess differences in metabolite profiles. Six insect-virulence compounds and one siderophore were mainly produced in OE*pks14*, as opposed to ∆*pks14*. The three MFs of insect-virulence compounds were an MF of BEA (*m/z* 784.4120, calcd. For C_45_H_57_N_3_O_9_H^+^, Δ−6.75 ppm) and BEA A/F (*m/z* 798.4277, calcd. For C_46_H_59_N_3_O_9_H^+^, Δ−6.89 ppm), an MF of BAS (*m/z* 909.6098, calcd. For C_48_H_84_N_4_O_12_H^+^, Δ−7.26 ppm), and an MF of ENN A (*m/z* 682.4590, calcd. For C_36_H_63_N_3_O_9_H^+^, Δ−7.76 ppm) (Figure 4A). BEA A and BEA F have similar chemical formulas and molecular weights but are different in structure. BEA A contains a secondary butyl (s-Bu) group of isoleucine (Ile) at R_7_ instead of the isopropyl (i-Pr) group of valine (Val) in BEA F (Figure 3). Ile and Val have similar molecular weights, and their MS/MS spectra cannot be distinguished; therefore, *m/z* 798.4277 [M+H]^+^ could be BEA A or BEA F. Two singletons of insect-virulence compounds represented BEA B (*m/z* 812.4425, calcd. for C_47_H_61_N_3_O_9_H^+^, Δ−7.50 ppm) and BEA C (*m/z* 848.4398, calcd. for C_48_H_63_N_3_O_9_Na^+^, Δ−7.54 ppm). One siderophore MF was FER (*m/z* 771.2446, calcd. for C_28_H_44_FeN_9_O_13_H^+^, Δ−5.19 ppm) (Figure 4A). ENN A was uniquely found in OE*pks14* and not in ∆*pks14*. BEA, BEA A, BEA B, BEA C, and BAS were increased in OE*pks14* compared to ∆*pks14*, and there was no difference for FER (Figure 4A and Figure S5). MS/MS spectrum annotation of classified metabolites was performed based on their fragmentation pattern (Figure S6). MF and singleton metabolites of interest are summarized in Table 1.

For in vivo data, MFs from comparative positive-ion data for BAWs injected with OE*pks14* or ∆*pks14* were generated for samples from 3, 5, and 7 DPI. The results identified one MF of insect virulence factors, namely, BEA (*m/z* 784.4120, calcd. for C_45_H_57_N_3_O_9_H^+^, Δ−6.75 ppm) and BEA A/F (*m/z* 798.4278, calcd. for C_46_H_59_N_3_O_9_H^+^, Δ−6.76 ppm), and one singleton of the siderophore FER (*m/z* 771.2459, calcd. for C_28_H_44_FeN_9_O_13_H^+^, Δ−3.50 ppm). BEA and BEA A were detected at higher levels in BAWs injected with OE*pks14* than with ∆*pks14* throughout the infection and colonization periods (3, 5, and 7 DPI). FER was found at higher levels from OE*pks14* samples at mid- (5 DPI) and late-stage (7 DPI) infections compared to ∆*pks14* samples (Figure 4B and Figure S7). MS/MS spectrum annotation of classified metabolites was determined using their fragmentation pattern (Figure S8). Annotated metabolites of interest are summarized in Table 1, and their chromatographic peak areas summarized in Table S1.

Although insect-virulence metabolites were mainly produced by OE*pks14* both in culture and in vivo, ENN A, BEA B, BEA C, and BAS were exclusively found in cultured OE*pks14*.

### 3.4. Beauvericins and Ferricrocin Were Upregulated in OEpks15 In Vivo

**Figure 5:**
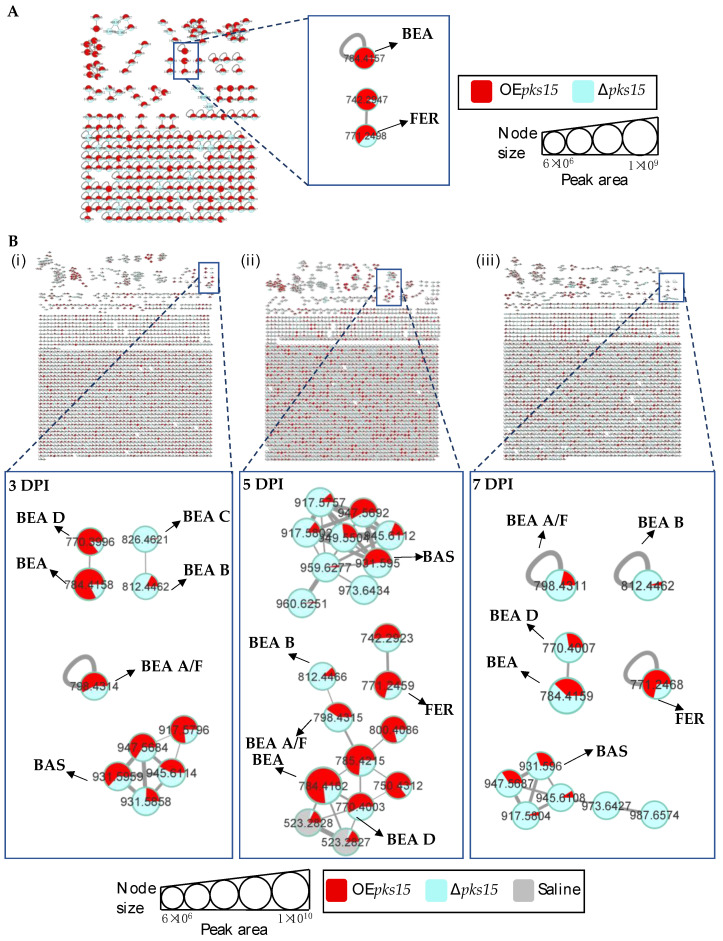
(**A**) Molecular networking of classified insect-virulence metabolites and a siderophore from OE*pks15* (red) and Δ*pks15* mutant (light blue) strains in culture identified beauvericin (BEA) and ferricrocin (FER). (**B**) Molecular networking of classified compounds from OE*pks15* (red) and Δ*pks15* mutant (light blue) strains in vivo at early-stage infection (3 DPI) for live larvae (**i**), mid-stage infection (5 DPI) for dead larvae (**ii**), and late-stage infection (7 DPI) for cadavers covered with fungal hyphae (**iii**) identified beauvericin (BEA), beauvericin A/F (BEA A/F), beauvericin B (BEA B), beauvericin C (BEA C), beauvericin D (BEA D), bassianolide (BAS), and ferricrocin (FER). Saline-injected BAWs were used as controls (gray). Node sizes represent the sums of chromatographic peak areas, and pie charts indicate chromatographic peak-area proportions for the detected insect virulence factors.

Next, insect-virulence metabolite profiles were compared for OE*pks15* and ∆*pks15* strains in culture. MS/MS spectral annotation of these compounds was determined using their fragmentation patterns (Figure S10). Positive ion data revealed increased levels of one singleton, BEA (*m/z* 784.4157, calcd. for C_45_H_57_N_3_O_9_H^+^, Δ−2.04 ppm), and one MF, FER (*m/z* 771.2498, calcd. for C_28_H_44_FeN_9_O_13_H^+^, Δ−1.56 ppm) for OE*pks15* compared to ∆*pks15* (Figure 5A and Figure S9).

In vivo comparative metabolite profiles for OE*pks15* and ∆*pks15* strains were also analyzed. The results identified seven insect-virulence metabolites, including BEA, BEA A, BEA B, BEA C, BEA D, ENN A, and BAS and the siderophore FER from the positive-ion data.

BEA (*m/z* 784.4157, calcd. for C_45_H_57_N_3_O_9_H^+^, Δ−2.04 ppm), BEA A/F (*m/z* 798.4305, calcd. for C_46_H_59_N_3_O_9_H^+^, Δ−3.38 ppm), BEA D (*m/z* 770.4002, calcd. for C_44_H_55_N_3_O_9_H+, Δ−1.94 ppm), and BAS (*m/z* 931.5959, calcd. for C_48_H_84_N_4_O_12_Na^+^, Δ−2.58 ppm) were upregulated in samples from BAWs injected with OE*pks15* at 3 and 5 DPI, except for BEA A/F, which was decreased at 5 DPI. All these insect-virulence compounds were subsequently reduced at 7 DPI in cadavers covered with hyphae (Figure 5B and Figure S11).

In BAWs injected with OE*pks15*, BEA B (*m/z* 812.4464, calcd. for C_47_H_61_N_3_O_9_H^+^, Δ−2.70 ppm) decreased throughout the experimental period, whereas BEA C (*m/z* 826.4626, calcd. for C_48_H_63_N_3_O_9_H^+^, Δ−2.05 ppm) was exclusively detected at 3 DPI (Figure 5B and Figure S11). FER (*m/z* 771.2498, calcd. for C_28_H_44_FeN_9_O_13_H^+^, Δ1.55 ppm) increased at 5 and 7 DPI (Figure 5B and Figure S11). MS/MS spectral annotation of these compounds was performed using their fragmentation patterns (Figure S12). Annotated metabolites in the MF and singletons, their MS/MS spectra, and chromatographic peak areas are summarized in Table 2 and Table S2. In summary, OE*pks15* mainly produced insect-virulence metabolites and ferricrocin in vivo rather than in culture.

### 3.5. pks14 Overexpression in Culture and pks15 Overexpression In Vivo Strongly Stimulated Insect-Virulence Metabolite Production

**Figure 6:**
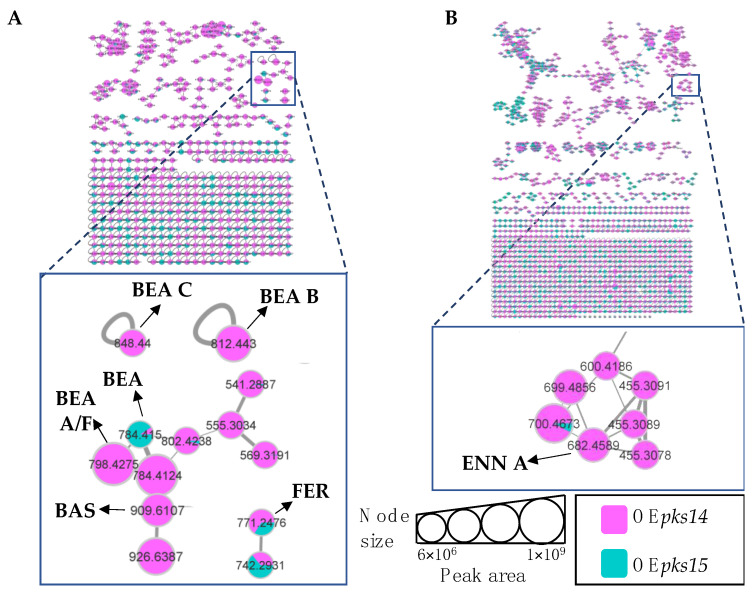
Molecular networking of insect-virulence metabolites and a siderophore from OE*pks14* (pink) and OE*pks15* (green) strains in culture identified beauvericin (BEA) and ferricrocin (FER) from both strains. (**A**) Beauvericin A/F (BEA A/F), beauvericin B (BEA B), beauvericin C (BEA C), and bassianolide (BAS) were found exclusively from OE*pks14* cells. (**B**) Enniatin A (ENN A) was found exclusively in OE*pks14* culture broth. Node sizes represent the sums of chromatographic peak areas, and pie charts indicate chromatographic peak-area proportions for the detected insect virulence factors.

**Figure 7:**
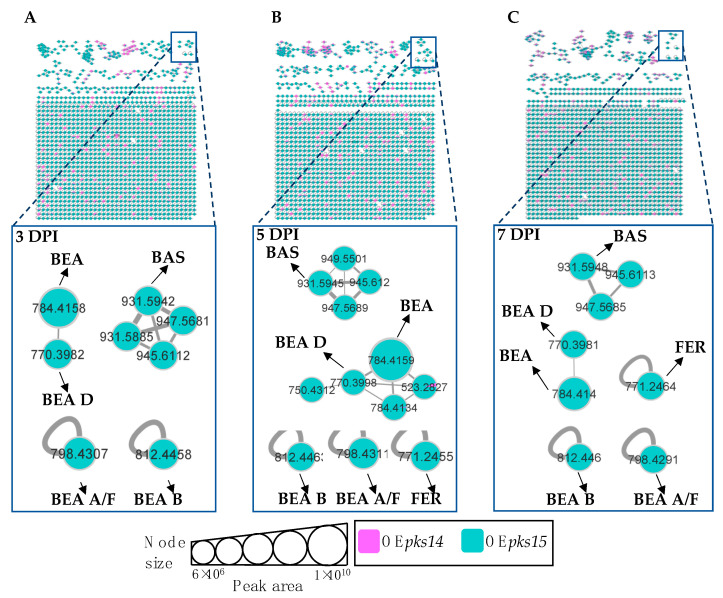
Molecular networking of insect-virulence metabolites and a siderophore for OE*pks14* (pink) and OE*pks15* (green) strains in vivo at (**A**) early-stage infection (3 DPI) for live larvae, (**B**) mid-stage infection (5 DPI) for dead larvae, and (**C**) late-stage infection (7 DPI) for cadavers covered with fungal hyphae identified beauvericin (BEA), beauvericin A/F (BEA A/F), beauvericin B (BEA B), beauvericin D (BEA D), bassianolide (BAS), and ferricrocin (FER) exclusively from OE*pks15*. Node sizes represent the sums of chromatographic peak areas, and pie charts indicate chromatographic peak area-proportions for the detected insect virulence factors.

Molecular networking comparisons between OE*pks14* and OE*pks15* strains in culture and in vivo were subsequently performed. In culture, expression of insect-virulence metabolites, including BEA A/F (*m/z* 798.4275, calcd. for C_46_H_59_N_3_O_9_H^+^, Δ−7.14 ppm), BEA B (*m/z* 812.4430, calcd. for C_47_H_61_N_3_O_9_H^+^, Δ−6.89 ppm), BEA C (*m/z* 848.4400, calcd. for C_48_H_63_N_3_O_9_Na^+^, Δ−7.31 ppm), BAS (*m/z* 909.6107, calcd. for C_48_H_84_N_4_O_12_H^+^, Δ−6.27 ppm), and ENN A (*m/z* 682.4598, calcd. for C_36_H_63_N_3_O_9_H^+^, Δ−6.59 ppm) were observed only for OE*pks14*, and FER levels were higher for OE*pks14* compared OE*pks15* (Figure 6). In contrast, in vivo comparison showed that several insect-virulence compounds were only detected from OE*pks15*-injected BAWs throughout the experimental period (3, 5, and 7 DPI). These compounds were BEA (*m/z* 784.4158, calcd. for C_45_H_57_N_3_O_9_H^+^, Δ−1.91 ppm), BEA A/F (*m/z* 798.4307, calcd. for C_46_H_59_N_3_O_9_H^+^, Δ−3.13 ppm), BEA B (*m/z* 812.4458, calcd. for C_47_H_61_N_3_O_9_H^+^, Δ−3.45 ppm), BEA D (*m/z* 770.3982, calcd. for C_44_H_55_N_3_O_9_H^+^, Δ−4.54 ppm), FER (*m/z* 771.2464, calcd. for C_28_H_44_FeN_9_O_13_H^+^, Δ−2.85 ppm), and BAS (*m/z* 931.5942, calcd. for C_48_H_84_N_4_O_12_Na^+^, Δ−4.40 ppm) (Figure 7).

Together, these results clearly demonstrated that the expression of insect-virulence compounds, such as BEA, ENN A, and BAS, was induced in *pks*-overexpressing strains. These compounds were detected exclusively from OE*pks14* in culture and from OE*pks15* in vivo. These findings suggest that PKS14 and PKS15 and their metabolites play roles in the production of insect-virulence compounds under specific conditions.

### 3.6. pks14 and pks15 Promoters Share Motifs with Beauvericin and Bassianolide Gene Cluster Promoters

Given the induction of insect virulence factors in the presence of overexpressed PKS14 and PSK15, we performed a comparative analysis of the promoter sequences. Interestingly, some surprising similarities were observed between the *pks14* and *pks15* promoters and those of genes in the BEA and BAS biosynthetic clusters. The promoter of *pks14*, the main synthase gene in cluster 22 of the *B. bassiana* BCC 2660 genome, shares motifs with the promoters of BEA synthetase (CTcgcCAAcACaGGa.CTTGAaCA; uppecase letters indicate high (>90%) similarity, and lowercase letter indicate low (≤20%) similarity), a gene for a hypothetical protein in the BEA biosynthetic cluster (TCTCTTCaAGacgGaCC) (GenBank accession number XM_008604818.1), and BAS synthetase (CTaC..CGTC.GaGTGC..C) (Figure S13A). 

Similarly, the *pks15* promoter shares motifs with the promoters of BEA synthetase (TcGCAgAcCAa.aTCaTTCacTcagCcacaCATTCaTTCaTACaTa.CAaaCATaA), a gene for a hypothetical protein in BEA gene cluster (TTcCAcATCac.cAaCacTCATaCATcCaTTaaaTCAa.cCATaC) (GenBank accession number XM_008604825), BAS synthetase (TCATCAaTC.TaCATC.AgTCaT.CaTTcaTaCA), and a gene for a hypothetical protein in the BAS biosynthetic cluster (TATcaTCAcCaTgC.TTCaTCaaTTCAcTcaTTCaTacagACA) (GenBank accession number XM_008597724.1) (Figure S13B). These data suggest that PKS14 and PKS15 can engage in crosstalk with the BEA and BAS biosynthetic clusters.

## 4. Discussion

In the event of fungal infection and colonization of insect hosts, numerous fungal secondary metabolites and enzymes coordinate to attack hosts. Thus, for secondary metabolite biosynthesis, crosstalk between secondary metabolite clusters will be needed; however, such crosstalk has rarely been found in fungi due to their complexity. Here, we show that the two *B. bassiana* insect-virulence factors, PKS14 and PKS15 [19,20,21], were involved in fungal growth and development and secondary metabolite production. PKS14 and PKS15 affected conidial yield and insect virulence, which has also been shown in previous studies [19,20,21]. Moreover, PKS15 expression was found in pseudohyphae-like cells and conidia, a cell type previously observed to be affected by knocking out *pks15* [21]. The pseudohyphae form is important for host infection for several fungal species, mainly yeasts such as *Candida lusitaniae* [35] and *Saccharomyces cerevisiae* clinical isolates [36]. These results emphasize that PKS14, PKS15, and their products are associated with the pathogenicity of *B. bassiana*. In addition, these two insect-virulence factors orchestrated the production of various NRPs required for insect pathogenesis. Our data demonstrate that the *pks14* and *pks15* clusters or PKS14 and PKS15 themselves support and enhance the production of some compounds implicated in insect pathogenesis, including an intracellular siderophore, in *B. bassiana*. We used integrated metabolomic approaches of LC-MS, LC-MS/MS, and GNPS molecular networking. Metabolomic profiles from culture and in vivo sampling of OE*pks14*, ∆*pks14*, OE*pks15*, and ∆*pks15* were determined and compared. Notable detected compounds included some widely recognized insect-virulence metabolites such as BEAs, BAS, and ENN A, and the intracellular siderophore FER. For OE*pks14*, their production was induced in culture rather than in vivo. In contrast, OE*pks15* exhibited enhanced production in vivo throughout the experimental period rather than in culture. 

Differentially enhanced production of insect-virulence metabolites under certain conditions, namely, by OE*pks14* in culture and OE*pks15* in vivo, could be the result of stereoisomers. A difference between culture and in vivo results has been described previously for cytochrome P450 2D6 (CYP2D6) inhibition by bupropion [37,38]. Bupropion is a strong inhibitor of CYP2D6 in vivo, but a weak inhibitor of CYP2D6 in vitro. Bupropion is a mixture of R- and S-bupropion which has stereoselectivity in pharmacology. The difference in inhibitor activity between in vitro and in vivo was found to be due to differences in stereoselectivity. In vivo stereoselectivity for R-bupropion was higher than that for S-bupropion. Moreover, stereoselective downregulation of CYP2D6 expression was also found to be higher in vivo than in vitro [39]. Since PKS14 and PKS15 each exhibited a specific induction condition for metabolite production, in culture or in vivo, respectively, this induction may be due to the stereoisomers of inducers.

Noticeably, *pks14* and *pks15* overexpression in this study contributed to the induced or increased production of these insect-virulence compounds. This suggested that PKS14 and PKS15, or their metabolites, might be directly or indirectly associated with key pathways in insect pathogenesis of *B*. *bassiana*. The observed associations and co-regulation between secondary metabolites could be the result of crosstalk between biosynthetic clusters, as the promoters of *pks14* and *pks15* exhibit similarities to promoters of various genes in the BEA and BAS gene clusters. Crosstalk between different clusters of secondary metabolites has also been described for *A. nidulans* [40]. Overexpression of the regulatory gene *scpR* in the NRPS gene cluster induced production of the polyketide asperfuranone by ScpR binding to the promoter of *afoA*, a regulatory gene in the asperfuranone biosynthetic cluster which shares a motif with the promoter of *scpR* [40]. In another *Aspergillus* species, *A. fumigatus*, crosstalk between two adjacent clusters is mediated by two non-regulatory genes. The genes *psoF* (a putative dual-functional methyltransferase/monooxygenase) and *psoG* (a hypothetical protein), located in the fumagillin biosynthetic cluster, are crucial for biosynthesis of pseurotin, a secondary metabolite belonging to the adjacent cluster [41]. 

In addition, some PKSs are notable for stimulating other molecules or biological factors in fungi. *Dictyostelium discoideum* PKS1 has been shown to be involved in production of a signaling molecule, 4-methyl-5-pentylbenzene-1,3-diol (MPBD) [42]. MPBD induced spore development by triggering the release of phosphopeptide spore differentiation factor 1 and controlling cell aggregation by regulation of the cAMP signaling pathway during *Dictyostelium* development [42,43]. An *A*. *carbonarius* PKS (AcPKS) has demonstrated a contribution to the expression of the global transcription factor LaeA—playing a crucial role in the pathogenicity and regulation of mycotoxin biosynthesis in the fungus [44]. Additionally, loss of AcPKS reduced the *laeA* transcript level. Nevertheless, in this study, it was not demonstrated how PKS14 and PKS15 or their polyketides are associated with the production of other insect-virulence metabolites, particularly NRPs. Therefore, identification of polyketides synthesized by PKS14 and PKS15, and their mechanistic involvement in biosynthetic pathways of those NRPs, need to be studied. This could lead to better understanding of the crosstalk between PKS clusters and other secondary metabolite clusters.

## Figures and Tables

**Table 1:** Summary of classified insect-virulence metabolites and a siderophore identified from OE*pks14* and ∆*pks14* strains in culture and in vivo.

Compound	Chemical Formula	Theoretical *m/z*	Observed *m/z*	Adduct	RT (min)	*m/z* Error (ppm)	MS/MS Fragment
Enniatin A	C_36_H_63_N_3_O_9_	682.4643	682.4590	[M+H]^+^	6.03	−7.76	250.1410, 350.1932, 477.2928, 577.3452
Ferricrocin	C_28_H_44_FeN_9_O_13_	771.2486	771.2446	[M+H]^+^	1.35	−5.19	455.1114, 524.1331, 599.1656
Beauvericin	C_45_H_57_N_3_O_9_	784.4173	784.4120	[M+H]^+^	5.85	−6.75	262.1443, 362.1956, 523.2801, 623. 3325
Beauvericin A/F	C_46_H_59_N_3_O_9_	798.4332	798.4277	[M+H]^+^	6.00	−6.89	262.1443, 376.2125, 537.2969, 637.3496
Beauvericin B	C_47_H_61_N_3_O_9_	812.4486	812.4425	[M+H]^+^	6.15	−7.50	398.1926, 559.2763, 673.3438
Beauvericin C	C_48_H_63_N_3_O_9_	848.4462	848.4398	[M+Na]^+^	6.42	−7.54	412.2090, 573.2927, 687.3608
Bassianolide	C_48_H_84_N_4_O_12_	909.6164	909.6098	[M+H]^+^	6.43	−7.26	456.3088, 555.3586, 682.4649, 782.5096

**Table 2:** Summary of classified insect-virulence metabolites and a siderophore identified from OEpks*15* and ∆*pks15* strains in culture media and in vivo.

Compound	Chemical Formula	Theoretical *m/z*	Observed *m/z*	Adduct	RT (min)	*m/z* Error (ppm)	MS/MS Fragment
Ferricrocin	C_28_H_44_FeN_9_O_13_	771.2486	771.2498	[M+H]^+^	1.35	1.55	455.1108, 542.1455, 599.1639
Beauvericin	C_45_H_57_N_3_O_9_	784.4173	784.4157	[M+H]^+^	5.86	−2.04	262.1454, 362.1982, 523.2820, 623.3319
Beauvericin A/ F	C_46_H_59_N_3_O_9_	798.4332	798.4305	[M+H]^+^	6.14	−3.38	398.1949, 559.2782, 659.3314
Beauvericin B	C_47_H_61_N_3_O_9_	812.4486	812.4464	[M+H]^+^	6.34	−2.70	537.2957, 651.3608
Beauvericin C	C_48_H_63_N_3_O_9_	826.4643	826.4626	[M+H]^+^	6.50	−2.05	276.1596, 390.2290, 551.3116, 665.3816
Beauvericin D	C_44_H_55_N_3_O_9_	770.4017	770.4002	[M+H]^+^	5.85	−1.94	531.2462, 631.2984
Bassanolide	C_48_H_84_N_4_O_12_	931.5983	931.5959	[M+Na]^+^	6.42	−2.58	447.2940, 577.3474, 704.4473, 804.5002

## Data Availability

The raw data of LC-MS in this study are available on the MassIVE website (https://massive.ucsd.edu; accessed on 3 January 2023) with accession numbers MSV000090990 (*pks14-*overexpressing strain in the culture medium), MSV000090991 (*pks14-*knockout strain in the culture medium), MSV000090992 (*pks14-*overexpressing strain in vivo), MSV000090993 (*pks14-*knockout strain in vivo), MSV000090994 (saline for PKS14 in vivo), MSV000090995 (*pks15-*overexpressing strain in the culture medium), MSV000090996 (*pks15-*knockout strain in the culture medium), MSV000090997 (*pks15-*overexpressing strain in vivo), MSV000090998 (*pks15-*knockout strain in vivo), and MSV000090999 (saline for PKS15 in vivo).

## Supplementary Materials

The following supporting information can be downloaded at: https://www.mdpi.com/article/10.3390/metabo13030425/s1. Table S1: Summary of chromatographic peak area of classified insect-virulence metabolites and a siderophore identified from OEpks*14* in culture and in vivo. Table S2: Summary of chromatographic peak area of classified insect-virulence metabolites and a siderophore identified from OEpks*15* in culture and in vivo. Figure S1: Molecular networking of classified metabolites for OE*pks14* (red) and Δ*pks14* (light blue) strains in (A) culture cells and (B) culture broth. Figure S2: Molecular networking of classified metabolites in OE*pks14* (red) and Δ*pks14* (light blue) strains in vivo at (A) early-stage infection (3 DPI) for live larvae, (B) mid-stage infection (5 DPI) for dead larvae, and (C) late-stage infection (7 DPI) for cadavers covered with fungal hyphae. Saline-injected BAWs were used as controls (gray). Figure S3: Molecular networking of classified metabolites for OE*pks15* (red) and Δ*pks15* (light blue) strains in culture. Figure S4: Molecular networking of classified metabolites for OE*pks15* (red) and Δ*pks15* (light blue) strains in vivo at (A) early-stage infection (3 DPI) for live larvae, (B) mid-stage infection (5 DPI) for dead larvae, and (C) late-stage infection (7 DPI) for cadavers covered with fungal hyphae. Saline-injected BAWs were used as controls (gray). Figure S5: Comparison of full insecticides and siderophore MS profiles between OE*pks14* and Δ*pks14* in culture revealed upregulation of beauvericin (BEA), beauvericin A/F (BEA A/F), beauvericin B (BEA B), beauvericin C (BEA C), bassianolide (BAS), and enniatin A (ENN A) in OE*pks14* compared to Δ*pks14*, whereas no difference was seen for ferricrocin (FER). Figure S6: MS/MS spectra of (A) beauvericin (BEA), (B) beauvericin A/F (BEA A/F), (C) beauvericin B (BEA B), (D) beauvericin C (BEA C), (E) enniatin A (ENN A), (F) bassianolide (BAS), and (G) ferricrocin (FER) from OE*pks14* and Δ*pks14* in culture. Hiv = 2-hydroxyisovaleric acid, NMePhe = N-methylphenylalanine, HMVA= 2-hydroxy-3methylvaleric acid, NMeIle= N-methylisoleucine, NMeLeu= N-methylleucine, Gly = glycine, and L-Ser = L-serine. Figure S7: Comparison of full insecticides and siderophore MS profiles between OE*pks14* and Δ*pks14* strains in vivo at early-stage infection (3 DPI) for live larvae, mid-stage infection (5 DPI) for dead larvae, and late-stage infection (7 DPI) for cadavers covered with fungal hyphae. Beauvericin (BEA), beauvericin A/F (BEA A/F), and ferricrocin (FER) were upregulated in OE*pks14* compared to Δ*pks14*. Figure S8: MS/MS spectra of (A) beauvericin (BEA), (B) beauvericin A/F (BEA A/F), and (C) ferricrocin (FER) from OE*pks14* in vivo at early-stage infection (3 DPI) for live larvae, mid-stage infection (5 DPI) for dead larvae, and late-stage infection (7 DPI) for cadavers covered with fungal hyphae. Hiv = 2-hydroxyisovaleric acid, NMePhe = N-methylphenylalanine, HMVA= 2-hydroxy-3methylvaleric acid, Gly = glycine, and L-Ser = L-serine. Figure S9: Comparison of insecticide and siderophore MS profiles between OE*pks15* and Δ*pks15* in culture revealed upregulation of beauvericin (BEA) and ferricrocin (FER) in OE*pks15* compared to Δ*pks15*. Figure S10: MS/MS spectra of (A) beauvericin (BEA) and (B) ferricrocin (FER) from OE*pks15* and Δ*pks15* in culture. Hiv = 2-hydroxyisovaleric acid, NMePhe = N-methylphenylalanine, Gly = glycine, and L-Ser = L-serine. Figure S11: Comparison of insecticide and siderophore MS profiles between OE*pks15* and Δ*pks15* in vivo at early-stage infection (3 DPI) for live larvae, mid-stage infection (5 DPI) for dead larvae, and late-stage infection (7 DPI) for cadavers covered with fungal hyphae. (A) Beauvericin (BEA), beauvericin A/F (BEA A/F), beauvericin B (BEA B), beauvericin C (BEA C), beauvericin D (BEA D), (B) bassianolide (BAS), and (C) ferricrocin (FER) were identified. Figure S12: MS/MS spectra of (A) beauvericin (BEA), (B) beauvericin A/F (BEA A/F), (C) beauvericin B (BEA B), (E) beauvericin D (BEA D), (F) bassianolide (BAS), and (G) ferricrocin (FER) from OE*pks15* and (D) beauvericin C (BEA D) from Δ*pks15* in vivo at early-stage infection (3 DPI) for live larvae, mid-stage infection (5 DPI) for dead larvae, and late-stage infection (7 DPI) for cadavers covered with fungal hyphae. Hiv = 2-hydroxyisovaleric acid, NMePhe = N-methylphenylalanine, HMVA= 2-hydroxy-3methylvaleric acid, Gly = glycine, and L-Ser = L-serine. Figure S13. Similarities between the promoters of genes (A) *pks14* or (B) *pks15* with those of genes in the beauvericin and bassianolide biosynthetic clusters.
