# Supplementary material for: Metabolomic Analysis Demonstrates the Impacts of Polyketide Synthases PKS14 and PKS15 on the Production of Beauvericins, Bassianolide, Enniatin A, and Ferricrocin in Entomopathogen Beauveria bassiana
Source: Metabolites. 2023 Mar 14;13(3):425. doi: 10.3390/metabo13030425 (PMC10057652; doi:10.3390/metabo13030425)
Supplement: Supplementary file 1 [file metabolites-13-00425-s001.zip › metabolites-2174978-supplementary.pdf]

Supplementary information

Table S1. Summary of chromatographic peak area of classified insect-virulence metabolites and a siderophore identified from OEpks*14* in culture and *in vivo*

Compound	Observed *m/z*	Adduct	RT (min)	Peak area			
				Culture medium	3 DPI	5DPI	7 DPI
Enniatin A	682.4590	$[M+H]^+$	6.03	$4.35 \times 10^7 \pm 1.63 \times 10^6$	-	-	-
Ferricrocin	771.2446	$[M+H]^+$	1.35	$9.37 \times 10^5 \pm 1.00 \times 10^5$	-	$7.04 \times 10^5 \pm 5.10 \times 10^4$	$7.42 \times 10^5 \pm 1.84 \times 10^4$
Beauvericin	784.4120	$[M+H]^+$	5.85	$1.18 \times 10^8 \pm 1.20 \times 10^7$	$3.51 \times 10^6 \pm 6.07 \times 10^4$	$3.55 \times 10^6 \pm 3.22 \times 10^5$	$4.07 \times 10^6 \pm 9.37 \times 10^4$
Beauvericin A/F	798.4277	$[M+H]^+$	6.00	$1.25 \times 10^8 \pm 1.63 \times 10^7$	$2.77 \times 10^5 \pm 1.87 \times 10^3$	$4.11 \times 10^5 \pm 7.83 \times 10^4$	$4.58 \times 10^5 \pm 7.71 \times 10^3$
Beauvericin B	812.4425	$[M+H]^+$	6.15	$6.18 \times 10^7 \pm 2.47 \times 10^6$	-	-	-
Beauvericin C	848.4398	$[M+Na]^+$	6.42	$3.13 \times 10^6 \pm 2.91 \times 10^5$	-	-	-
Bassianolide	909.6098	$[M+H]^+$	6.43	$2.94 \times 10^7 \pm 9.90 \times 10^5$	-	-	-

Table S2. Summary of chromatographic peak area of classified insect-virulence metabolites and a siderophore identified from OEpks*15* in culture and *in vivo*

Compound	Observed *m/z*	Adduct	RT (min)	Peak area			
				Culture medium	3 DPI	5DPI	7 DPI
Ferricrocin	771.2498	$[M+H]^+$	1.35	$5.75 \times 10^5 \pm 2.74 \times 10^4$	-	$1.06 \times 10^8 \pm 1.41 \times 10^6$	$2.57 \times 10^8 \pm 7.78 \times 10^6$
Beauvericin	784.4157	$[M+H]^+$	5.86	$5.03 \times 10^5 \pm 5.87 \times 10^4$	$3.34 \times 10^9 \pm 6.36 \times 10^7$	$6.24 \times 10^9 \pm 6.51 \times 10^8$	$1.90 \times 10^9 \pm 4.17 \times 10^8$
Beauvericin A/ F	798.4305	$[M+H]^+$	6.14	-	$4.80 \times 10^8 \pm 1.46 \times 10^8$	$6.71 \times 10^8 \pm 7.42 \times 10^7$	$2.15 \times 10^8 \pm 5.66 \times 10^6$
Beauvericin B	812.4464	$[M+H]^+$	6.34	-	$1.33 \times 10^7 \pm 1.48 \times 10^6$	$3.42 \times 10^7 \pm 6.79 \times 10^6$	$1.70 \times 10^7 \pm 3.25 \times 10^6$
Beauvericin C	826.4626	$[M+H]^+$	6.50	-	$7.42 \times 10^4 \pm 3.27 \times 10^4$	-	-
Beauvericin D	770.4002	$[M+H]^+$	5.85	-	$2.26 \times 10^8 \pm 2.12 \times 10^6$	$1.77 \times 10^8 \pm 2.12 \times 10^7$	$8.25 \times 10^7 \pm 3.25 \times 10^6$
Bassanolide	931.5959	$[M+Na]^+$	6.42	-	$1.55 \times 10^8 \pm 2.47 \times 10^7$	$1.89 \times 10^8 \pm 0.00$	$7.27 \times 10^7 \pm 7.99 \times 10^6$

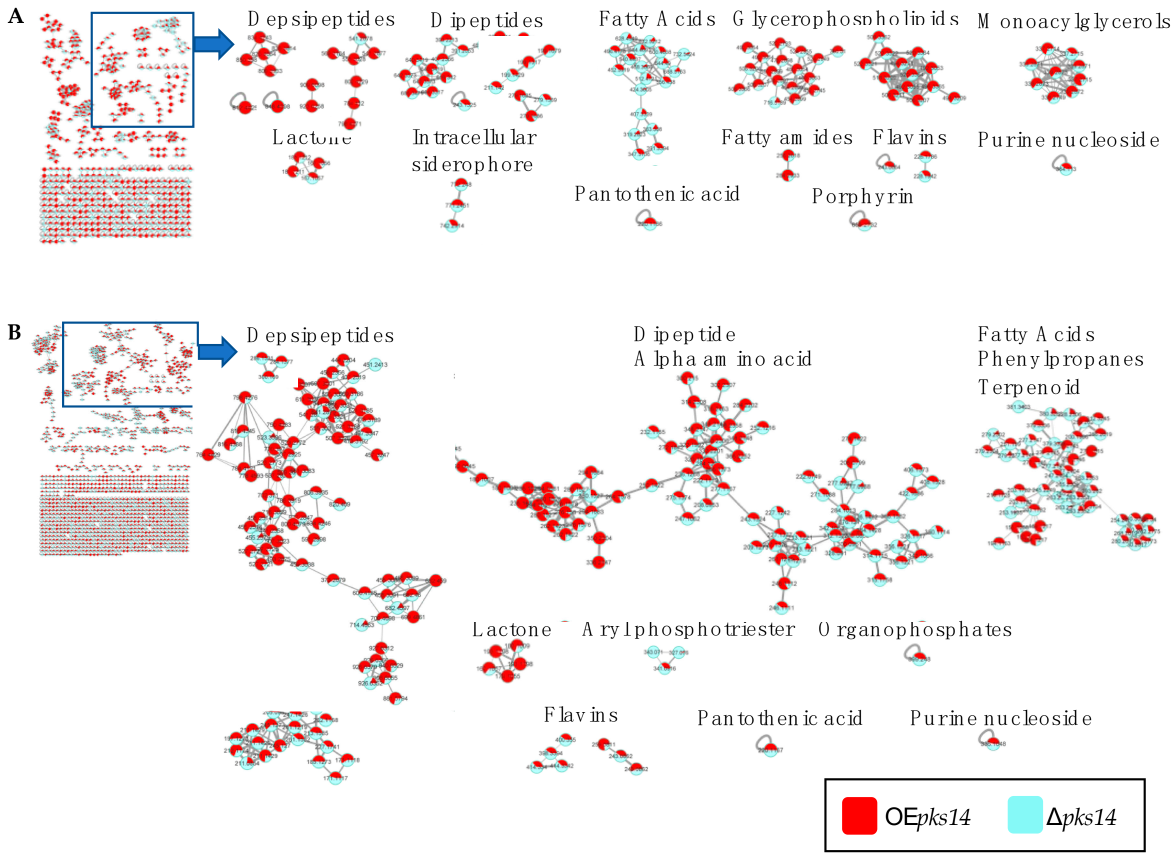

Figure S1. Molecular networking of classified metabolites for OE*pks14* (red) and Δ*pks14* (light blue) strains in (A) culture cells and (B) culture broth.

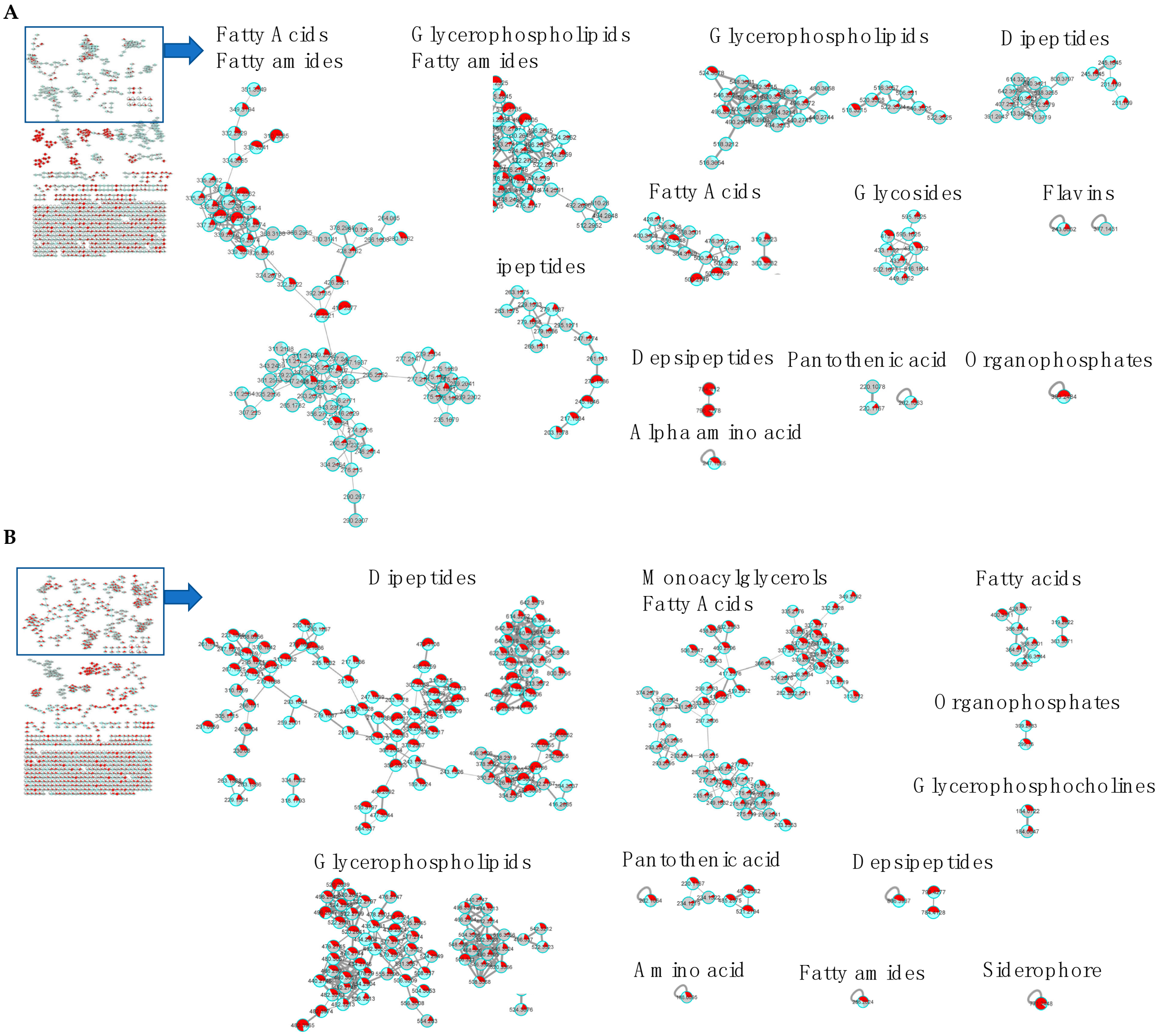

Figure S2. Molecular networking of classified metabolites in OE*pks14* (red) and Δ*pks14* (light blue) strains *in vivo* at (A) early-stage infection (3 DPI) for live larvae, (B) mid-stage infection (5 DPI) for dead larvae, and (C) late-stage infection (7 DPI) for cadavers covered with fungal hyphae. Saline-injected BAWs were used as controls (gray).

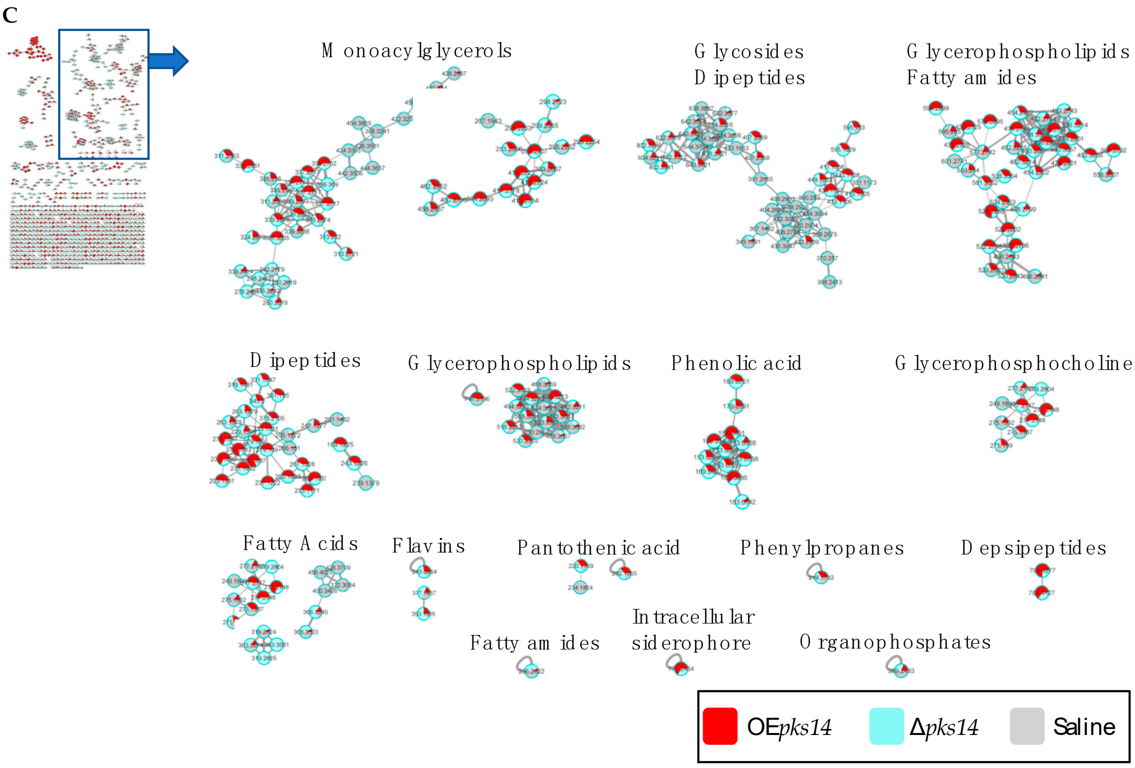

Figure S2. (continued) Molecular networking of classified metabolites in OE*pks14* (red) and Δ*pks14* (light blue) strains *in vivo* at (A) early-stage infection (3 DPI) for live larvae, (B) mid-stage infection (5 DPI) for dead larvae, and (C) late-stage infection (7 DPI) for cadavers covered with fungal hyphae. Saline-injected BAWs were used as controls (gray).

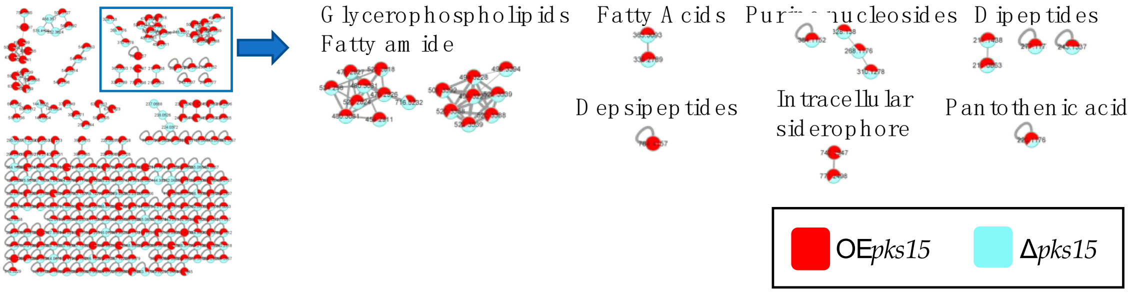

Figure S3. Molecular networking of classified metabolites for OE*pks15* (red) and Δ*pks15* (light blue) strains in culture.

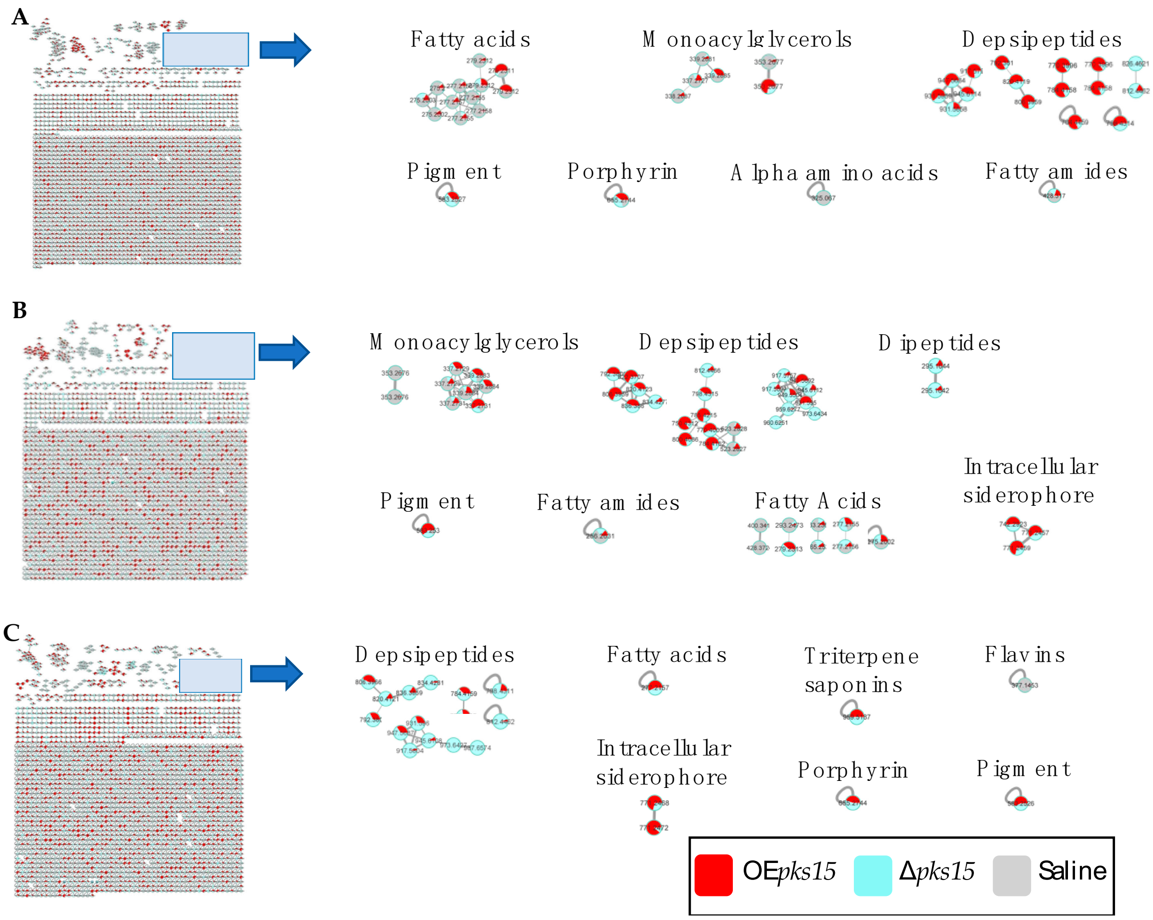

Figure S4. Molecular networking of classified metabolites for OE*pks15* (red) and Δ*pks15* (light blue) strains *in vivo* at (A) early-stage infection (3 DPI) for live larvae, (B) mid-stage infection (5 DPI) for dead larvae, and (C) late-stage infection (7 DPI) for cadavers covered with fungal hyphae. Saline-injected BAWs were used as controls (gray).

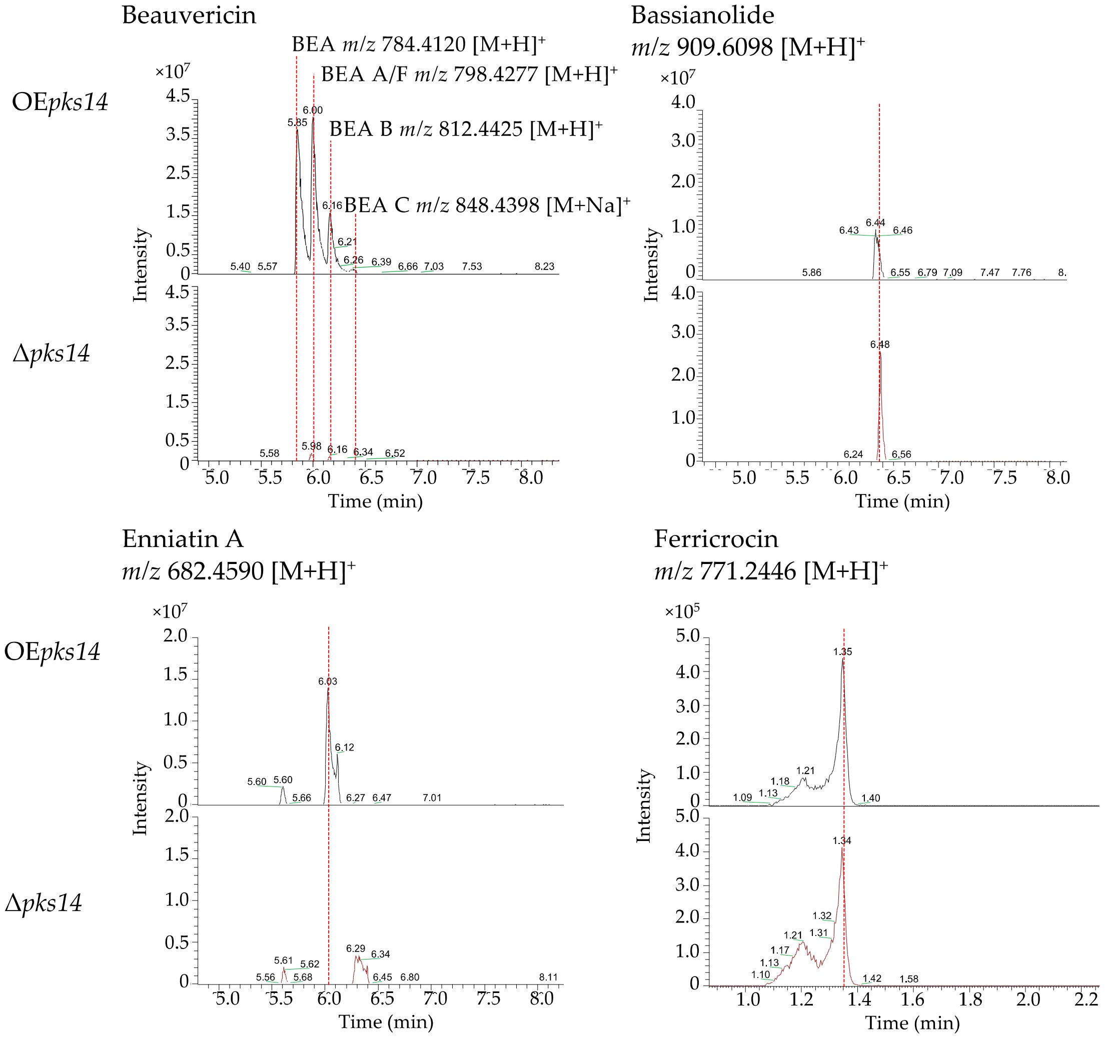

Figure S5. Comparison of full insecticides and siderophore MS profiles between OE*pks14* and Δ*pks14* in culture revealed up-regulation of beauvericin (BEA), beauvericin A/F (BEA A/F), beauvericin B (BEA B), beauvericin C (BEA C), bassianolide (BAS), and enniatin A (ENN A) in OE*pks14* compared to Δ*pks14* while no difference was seen for ferricrocin (FER).

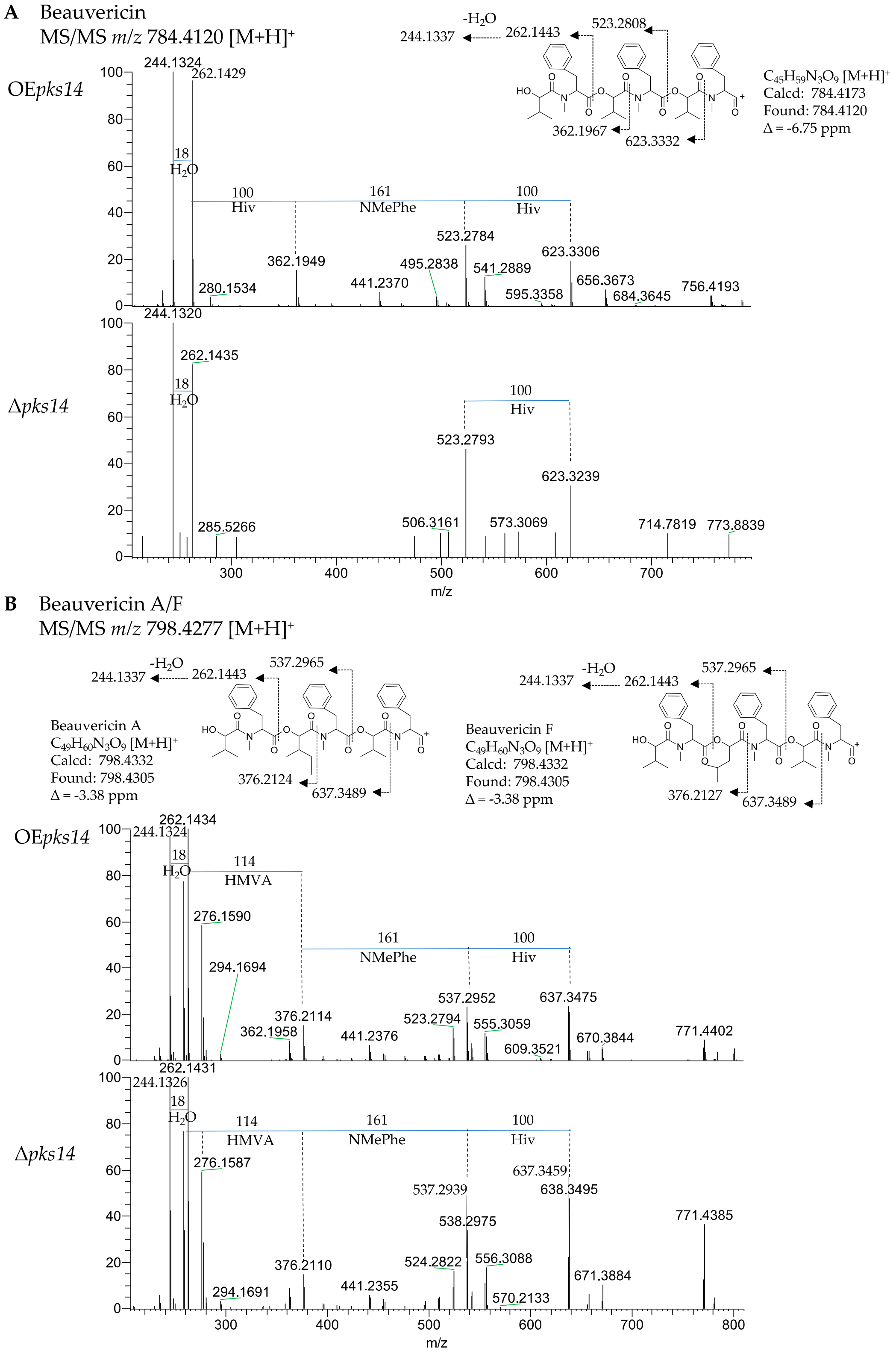

Figure S6. MS/MS spectra of (A) beauvericin (BEA), (B) beauvericin A/F (BEA A/F), (C) beauvericin B (BEA B), (D) beauvericin C (BEA C), (E) enniatin A (ENN A), (F) bassianolide (BAS), and (G) ferricrocin (FER) from OE*pks14* and Δ*pks14* in culture. Hiv = 2-hydroxyisovaleric acid, NMePhe = N-methylphenylalanine, HMVA= 2-hydroxy-3methylvaleric acid, NMeIle= N-methylisoleucine, NMeLeu= N-methylleucine, Gly = glycine, and L-Ser = L-serine.

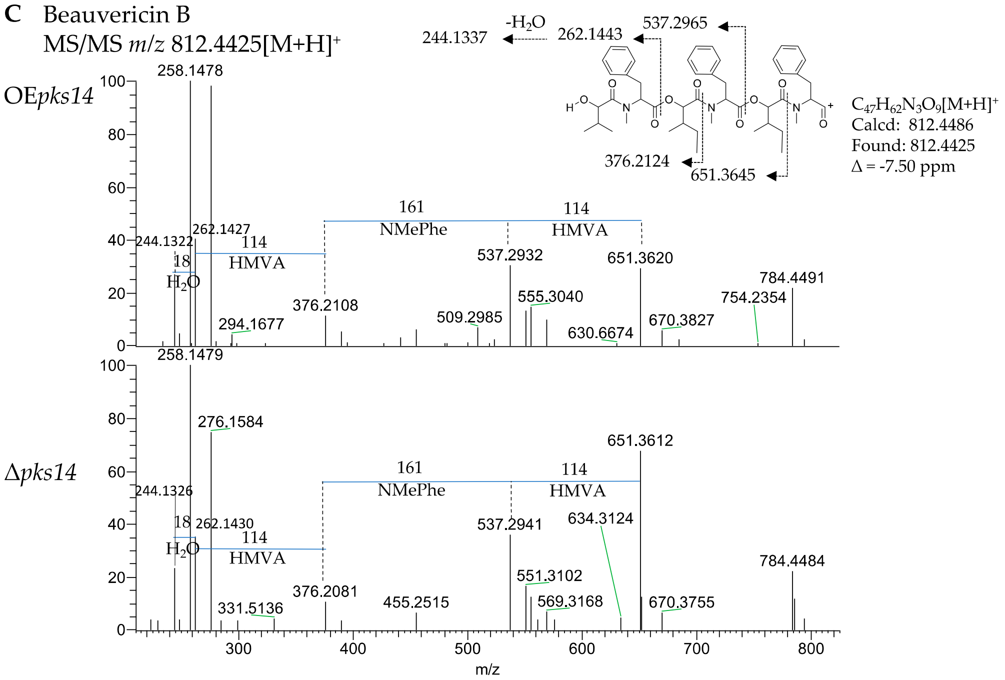

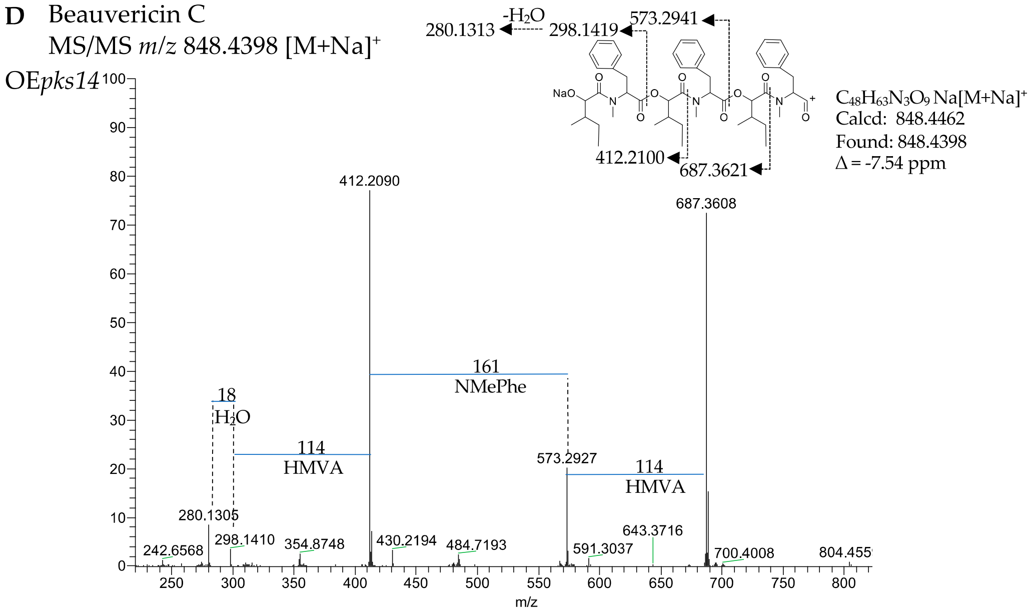

Figure S6. (continued)

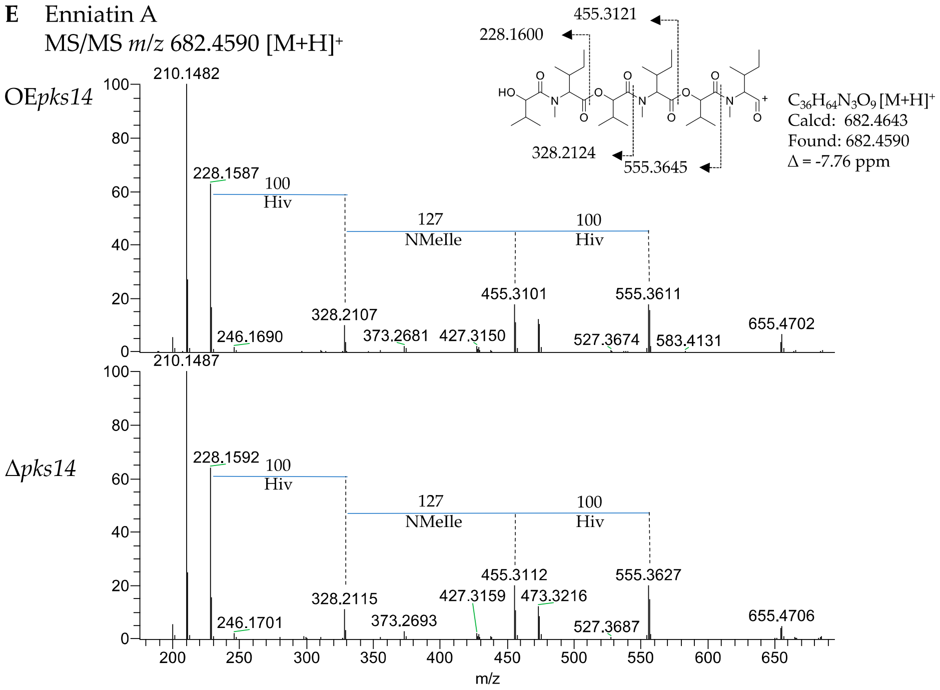

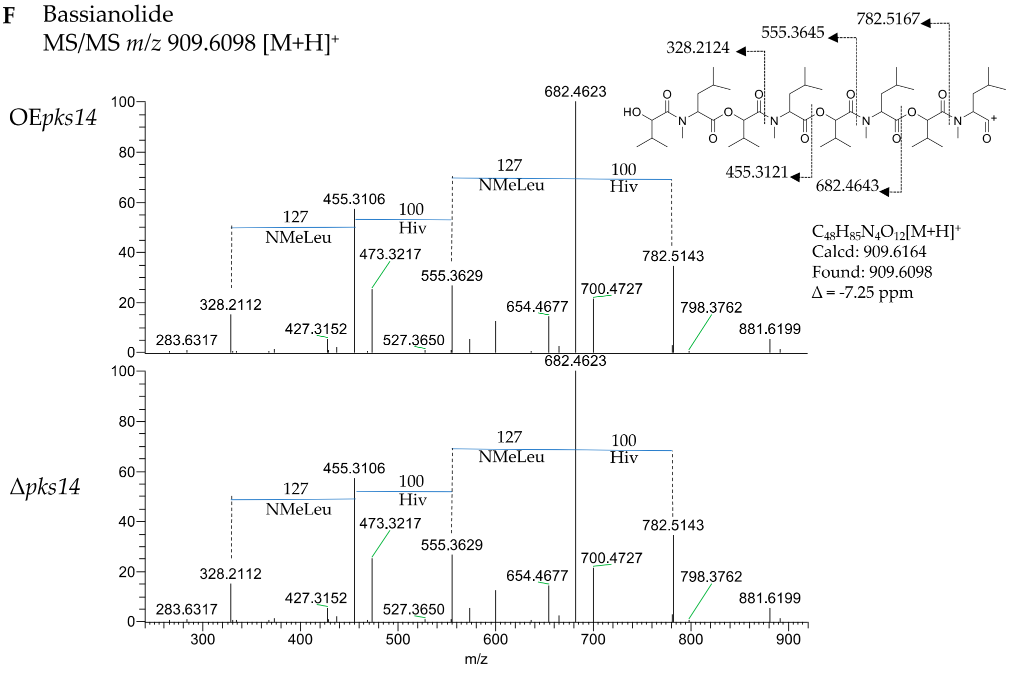

Figure S6. (continued)

G Ferricrocin

MS/MS *m/z* 771.2446 [M+H]+

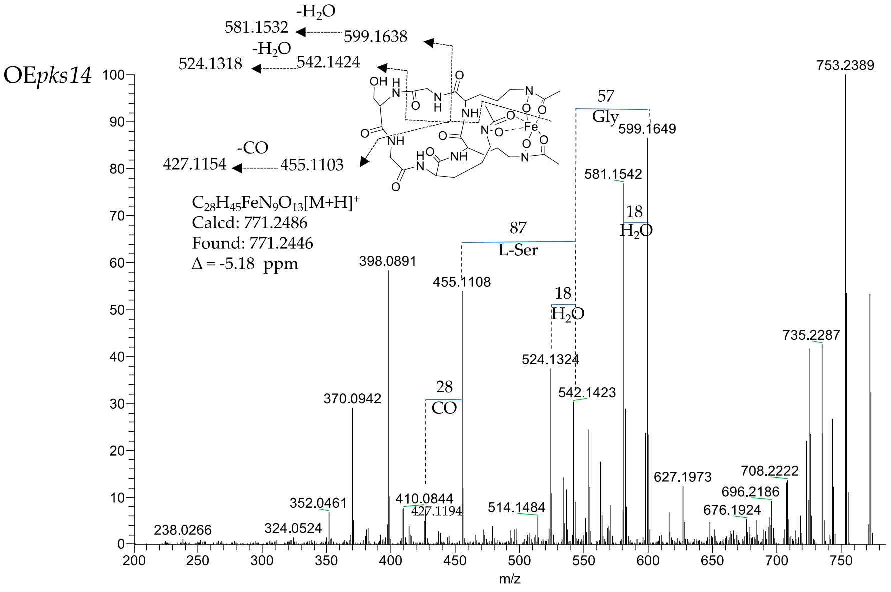

Figure S6. (continued)

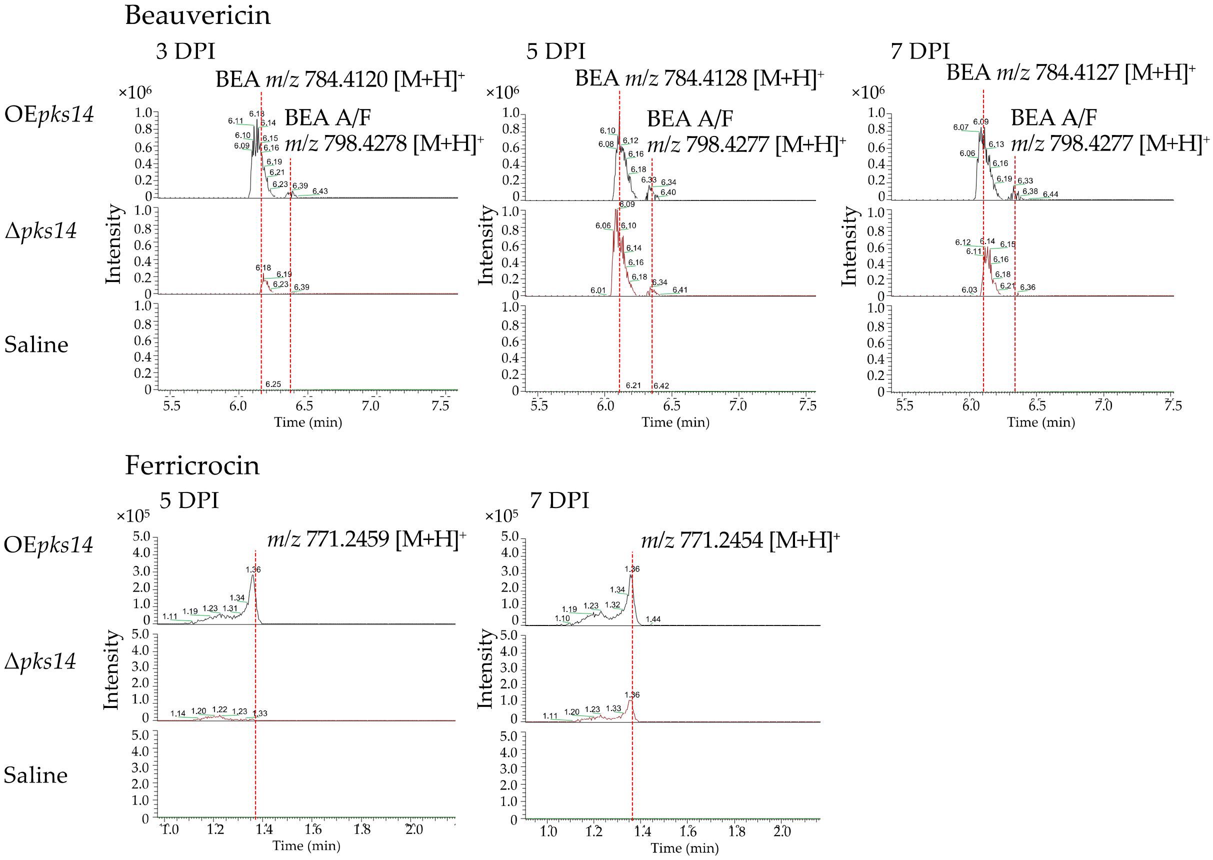

Figure S7. Comparison of full insecticides and siderophore MS profiles between OE*pks14* and Δ*pks14* strains *in vivo* at early-stage infection (3 DPI) for live larvae, mid-stage infection (5 DPI) for dead larvae, and late-stage infection (7 DPI) for cadavers covered with fungal hyphae. Beauvericin (BEA), beauvericin A/F (BEA A/F), and ferricrocin (FER) were up-regulated in OE*pks14* compared to Δ*pks14*.

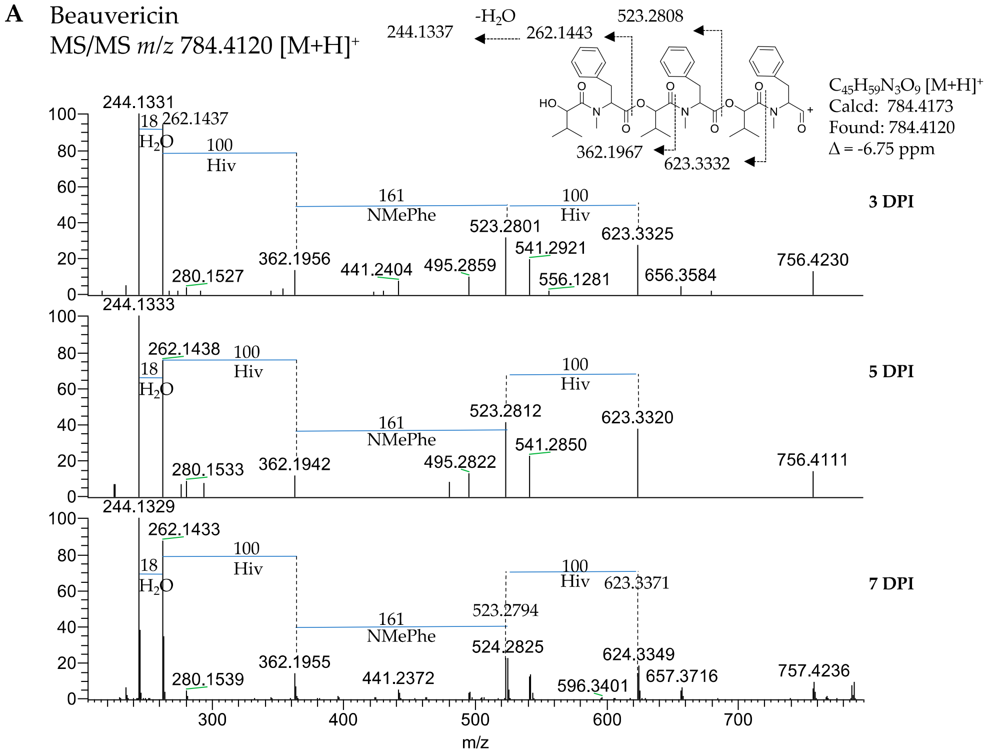

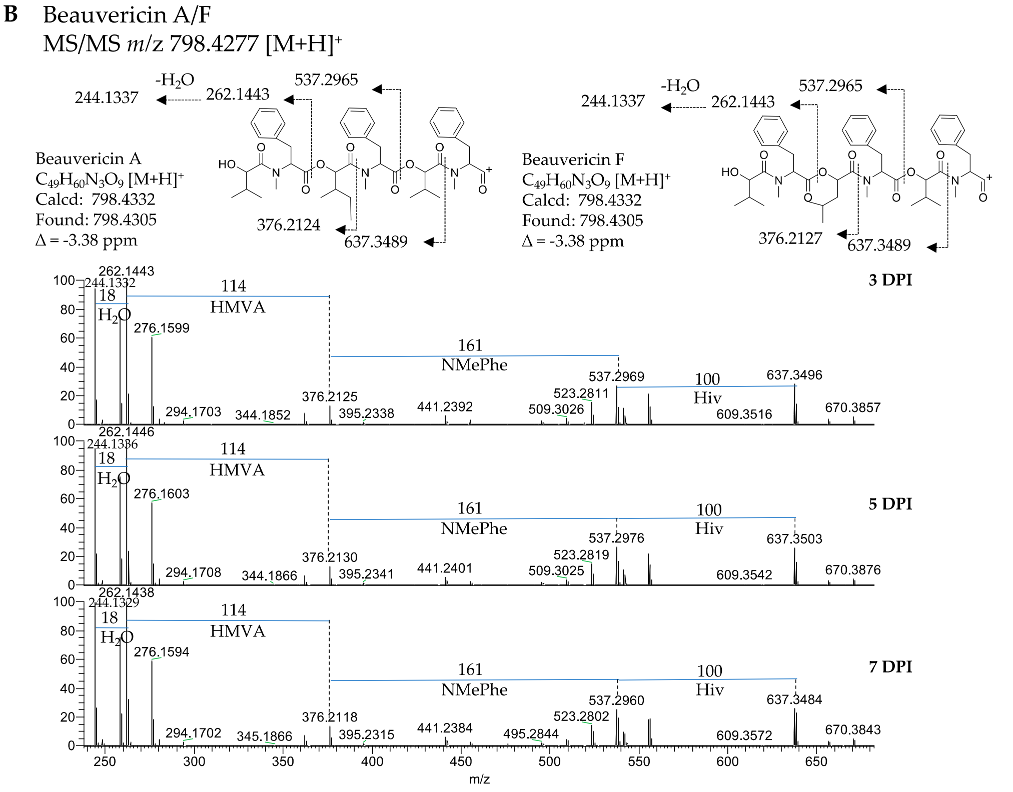

Figure S8. MS/MS spectra of (A) beauvericin (BEA), (B) beauvericin A/F (BEA A/F), and (C) ferricrocin (FER) from OE*pks14 in vivo* at early-stage infection (3 DPI) for live larvae, mid-stage infection (5 DPI) for dead larvae, and late-stage infection (7 DPI) for cadavers covered with fungal hyphae. Hiv = 2-hydroxyisovaleric acid, NMePhe = N-methylphenylalanine, HMVA= 2-hydroxy-3methylvaleric acid, Gly = glycine, and L-Ser = L-serine.

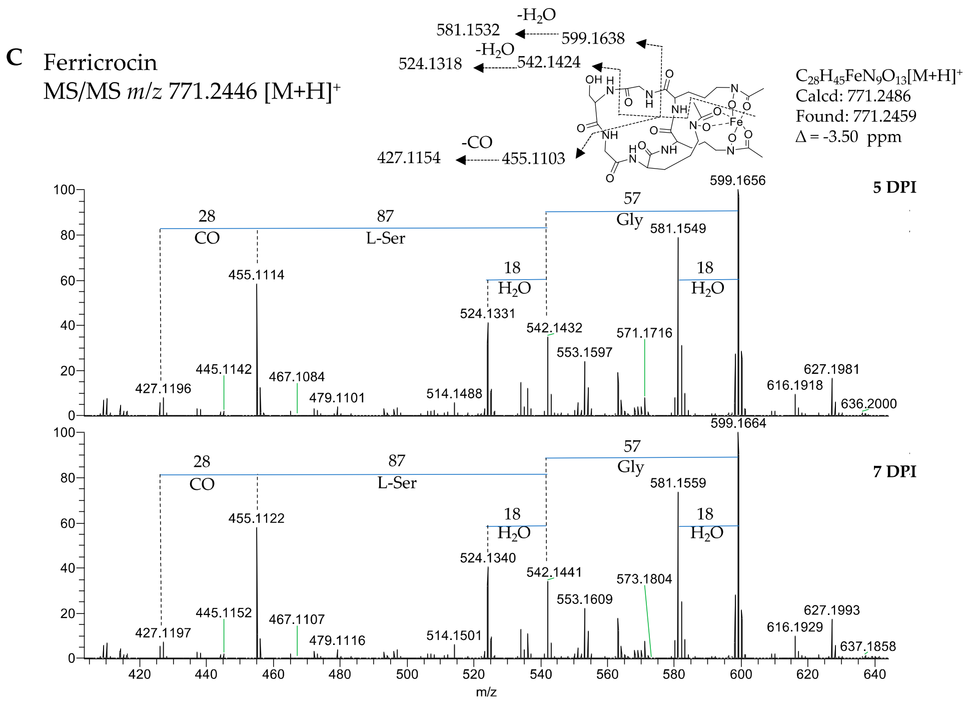

Figure S8. (continued)

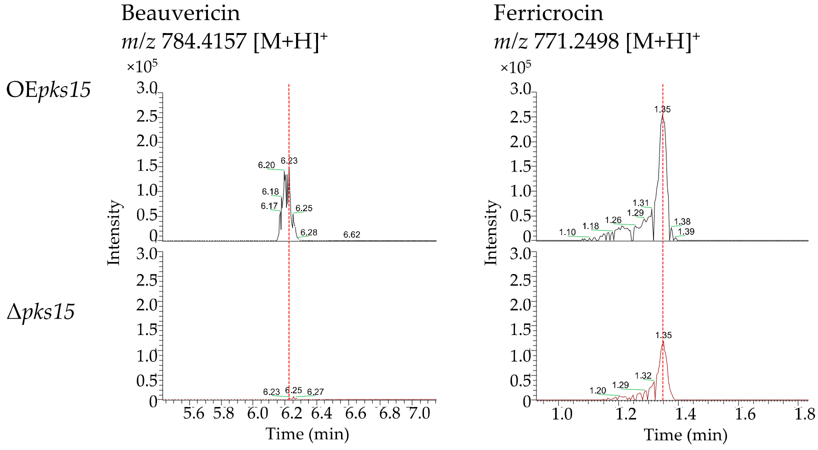

Figure S9. Comparison of insecticide and siderophore MS profiles between OE*pks15* and Δ*pks15* in culture revealed up-regulation of beauvericin (BEA) and ferricrocin (FER) in OE*pks15* compared to Δ*pks15*.

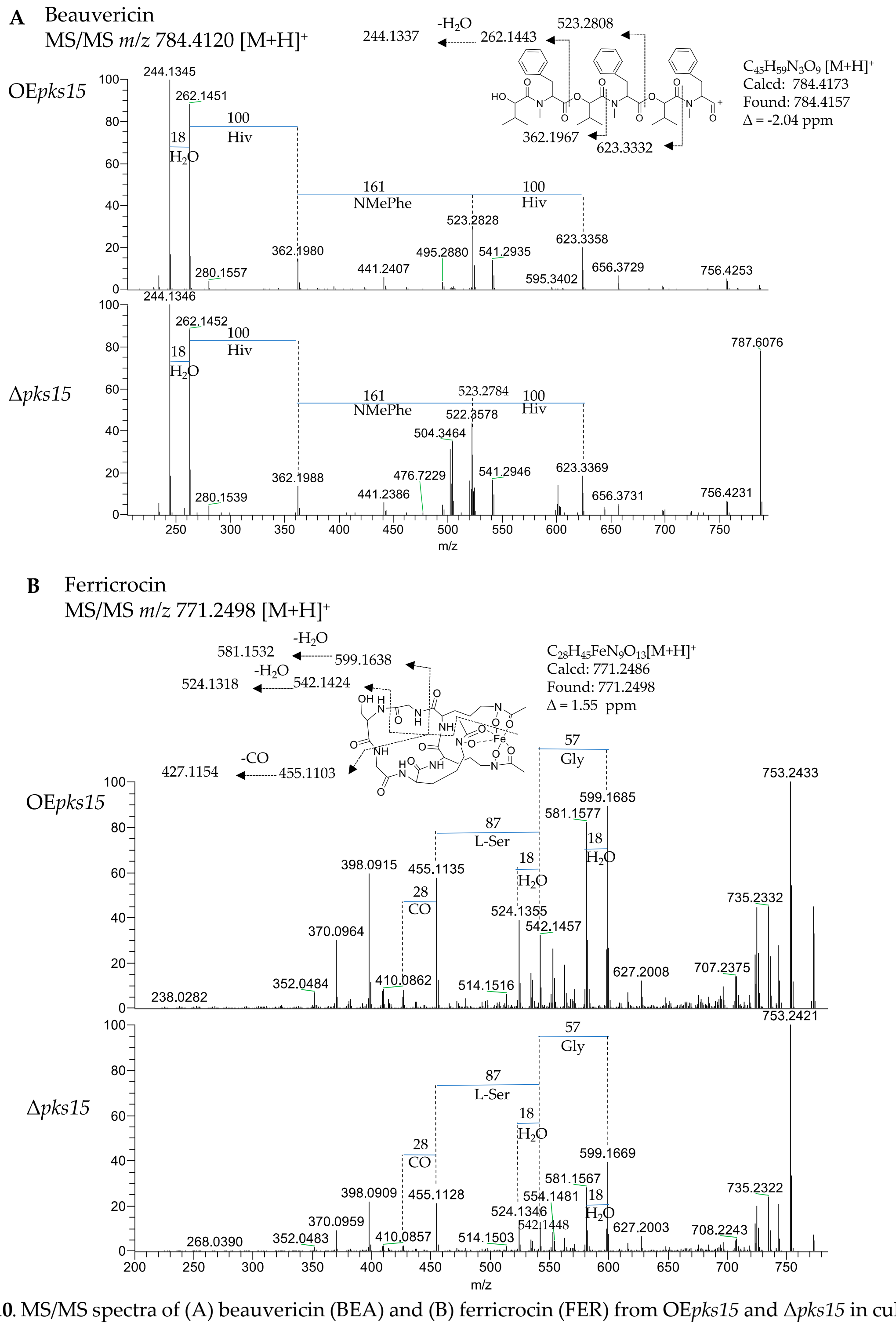

Figure S10. MS/MS spectra of (A) beauvericin (BEA) and (B) ferricrocin (FER) from OE*pks15* and Δ*pks15* in culture. Hiv = 2-hydroxyisovaleric acid, NMePhe = N-methylphenylalanine, Gly = glycine, and L-Ser = L-serine.

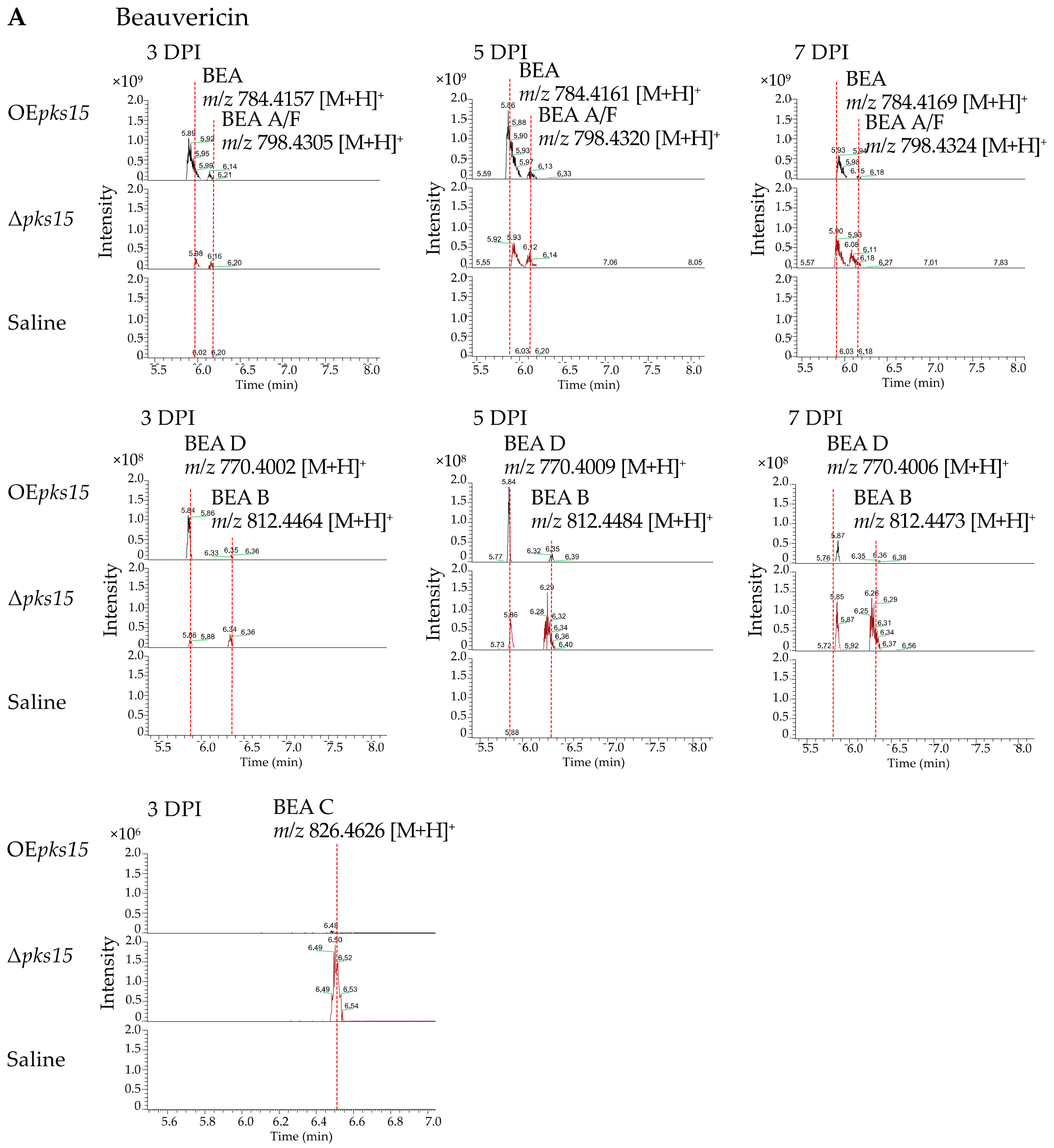

Figure S11. Comparison of insecticide and siderophore MS profiles between OE*pks15* and Δ*pks15 in vivo* at early-stage infection (3 DPI) for live larvae, mid-stage infection (5 DPI) for dead larvae, and late-stage infection (7 DPI) for cadavers covered with fungal hyphae. (A) Beauvericin (BEA), beauvericin A/F (BEA A/F), beauvericin B (BEA B), beauvericin C (BEA C), beauvericin D (BEA D), (B) bassianolide (BAS), and (C) ferricrocin (FER) were identified.

B Bassianolide

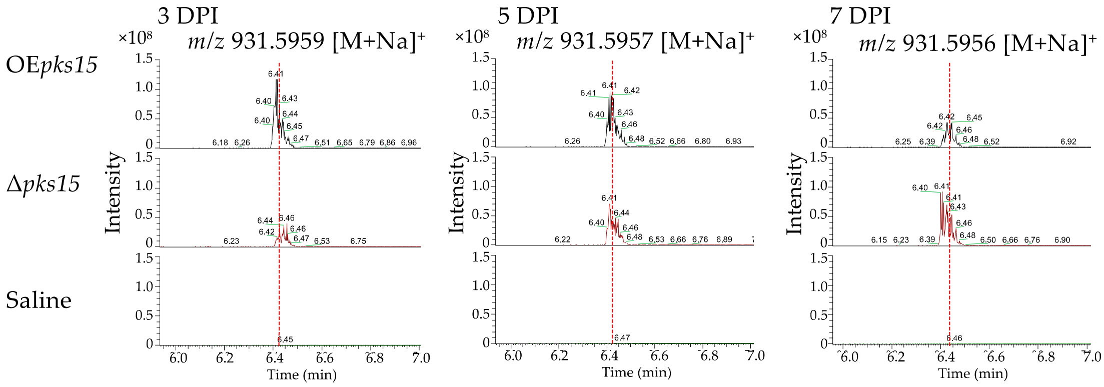

C Ferricrocin

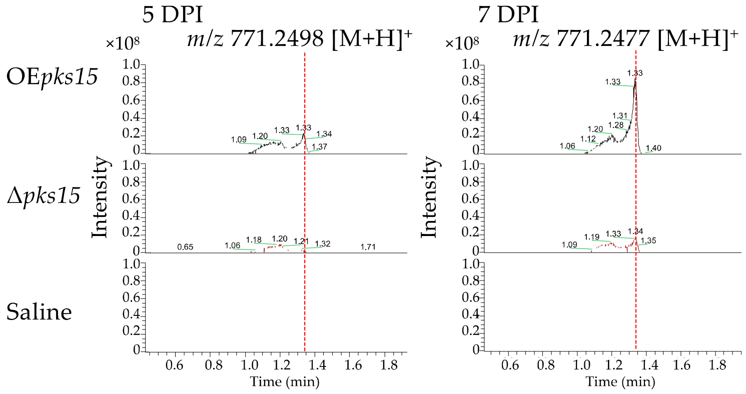

Figure S11. (continued)

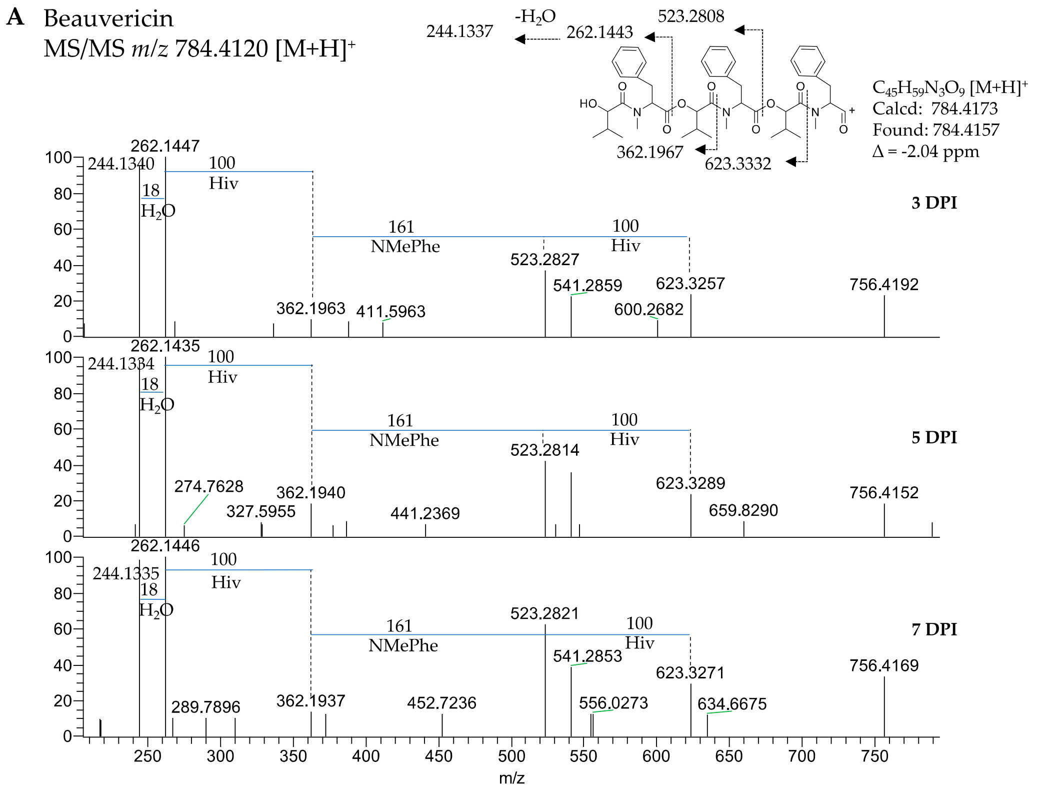

Figure S12. MS/MS spectra of (A) beauvericin (BEA), (B) beauvericin A/F (BEA A/F), (C) beauvericin B (BEA B), (E) beauvericin D (BEA D), (F) bassianolide (BAS), and (G) ferricrocin (FER) from OE*pks15* and (D) beauvericin C (BEA D) from Δ*pks15 in vivo* at early-stage infection (3 DPI) for live larvae, mid-stage infection (5 DPI) for dead larvae, and late-stage infection (7 DPI) for cadavers covered with fungal hyphae. Hiv = 2-hydroxyisovaleric acid, NMePhe = N-methylphenylalanine, HMVA= 2-hydroxy-3methylvaleric acid, Gly = glycine, and L-Ser = L-serine.

B Beauvericin A/F

MS/MS m/z 798.4277 $[M+H]^+$

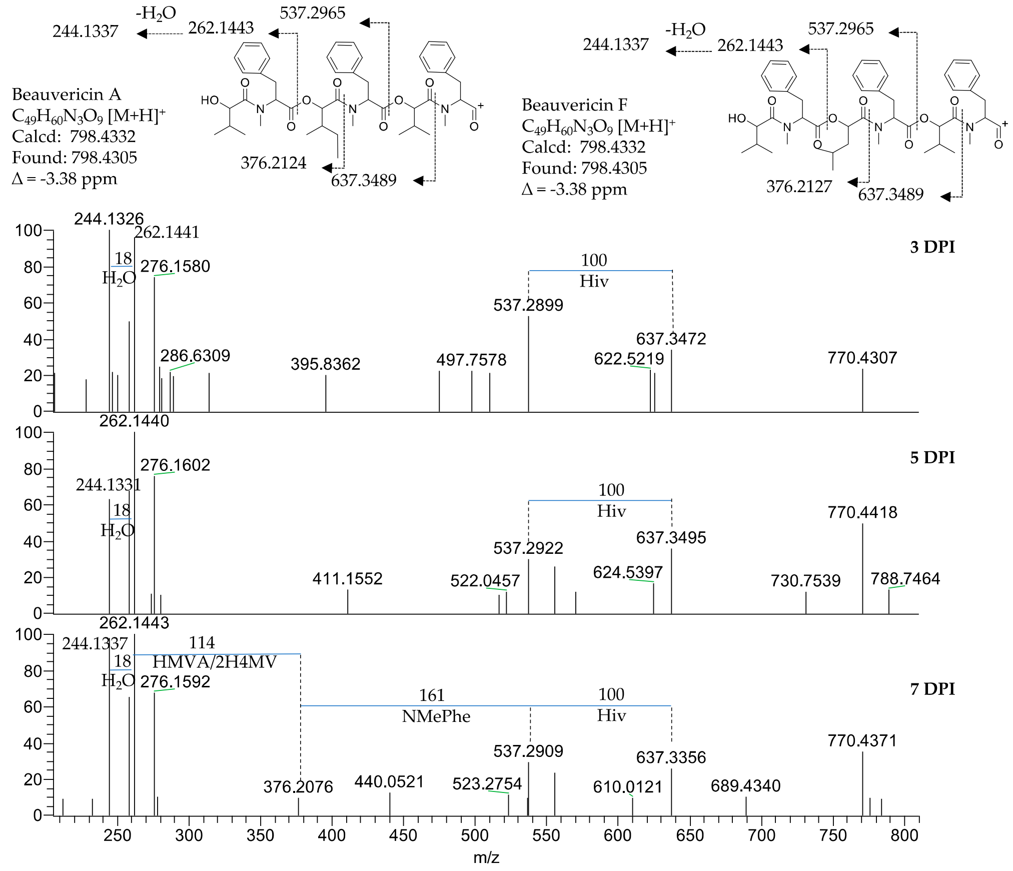

Figure S12. (continued)

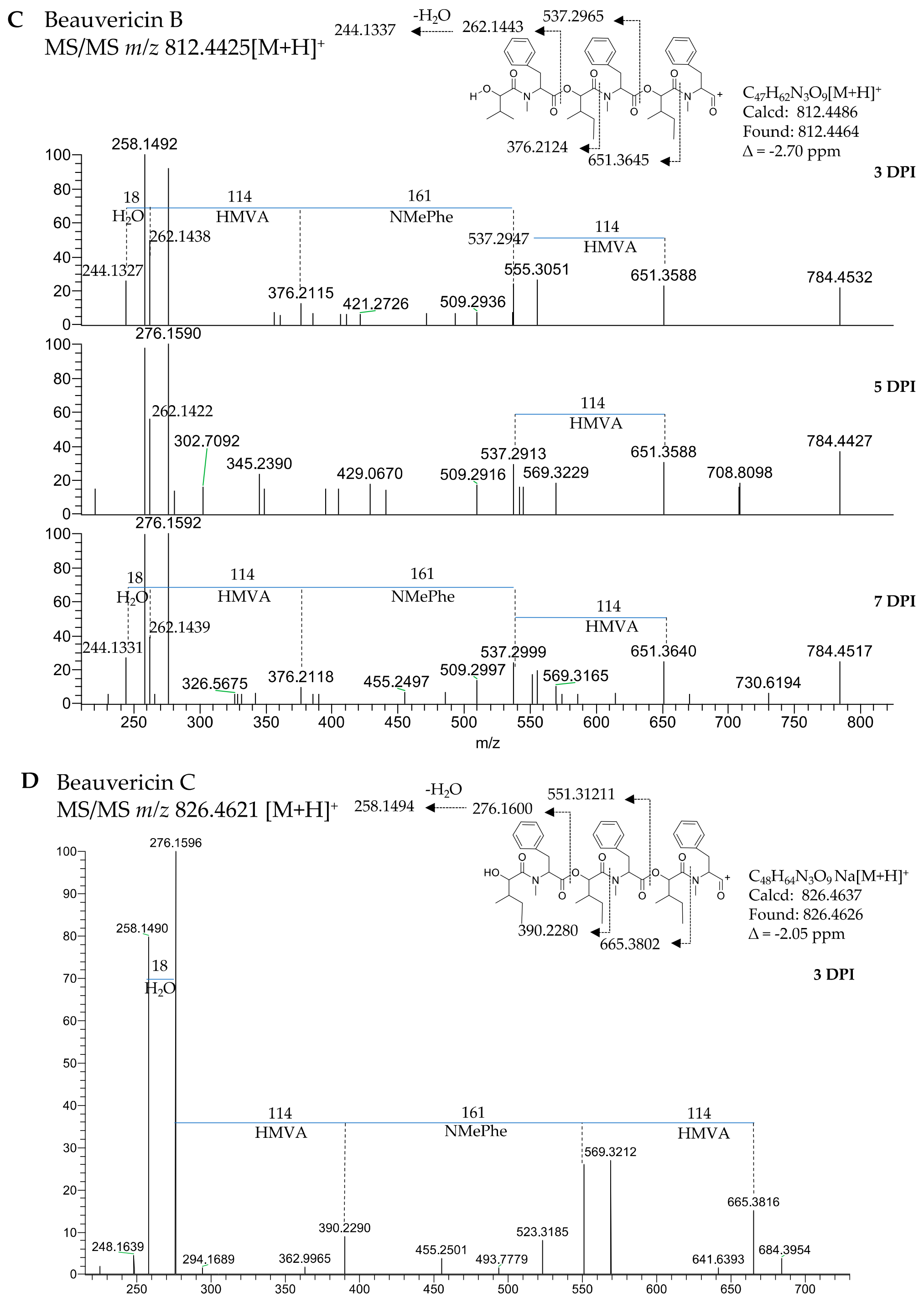

Figure S12. (continued)

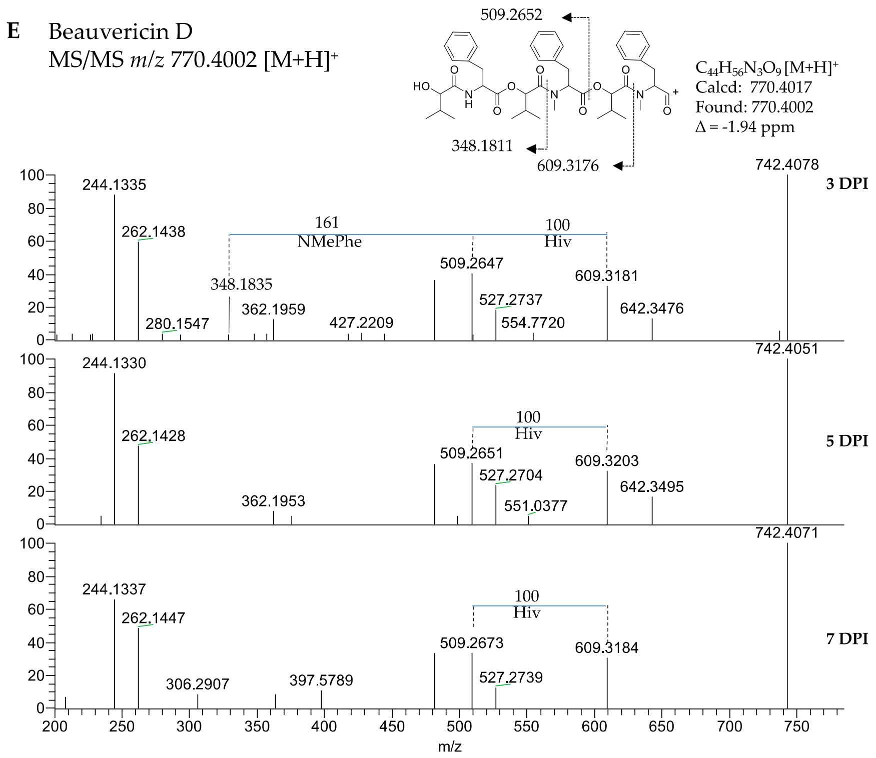

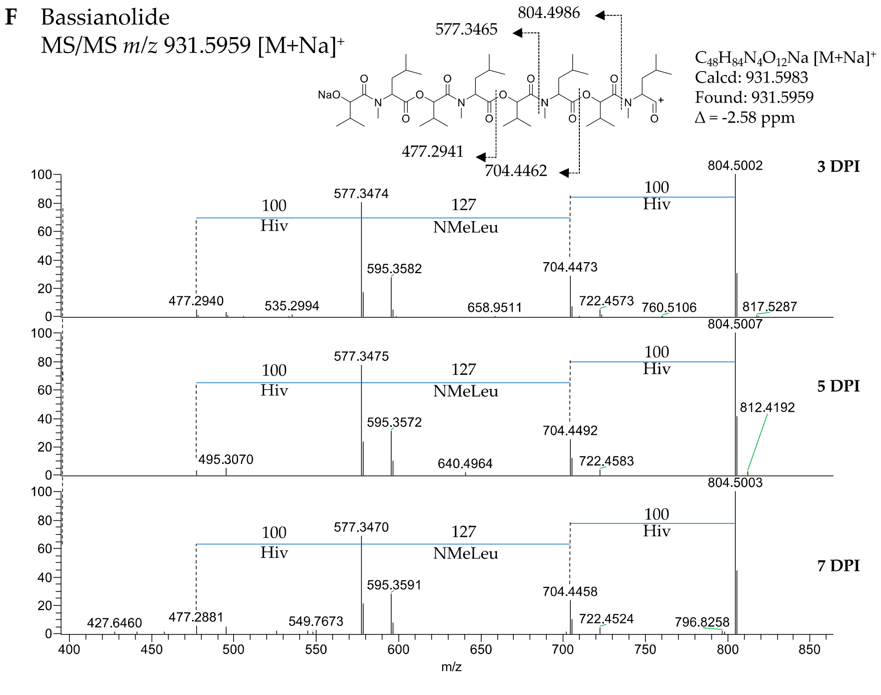

Figure S12. (continued)

G Ferricrocin

MS/MS m/z 771.2449 $[M+H]^+$

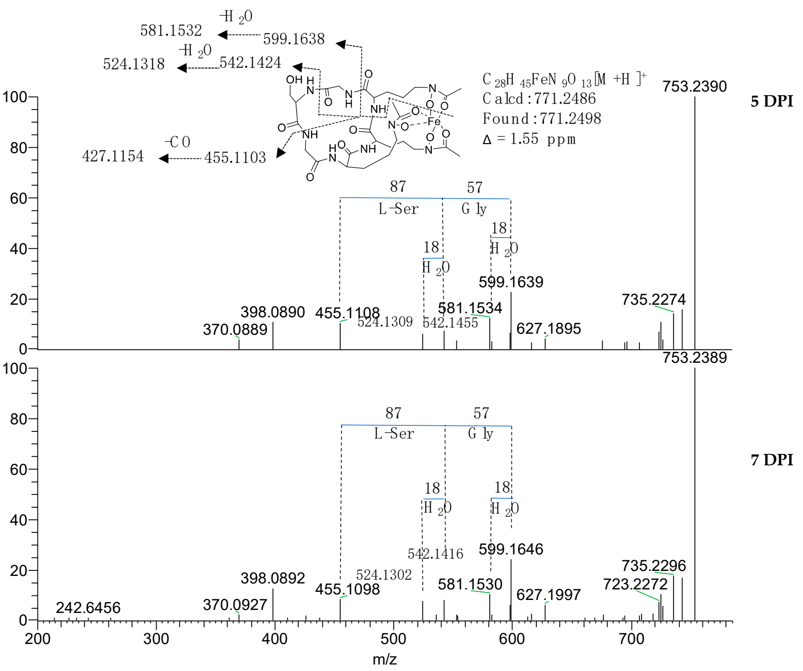

Figure S12. (continued)

A

```
PKS14           TCTTTATTAC TTGGCGTAAA CTCTCTTCAA GACGGGC.CT TGAACA.... ..GAGAG
beauvericin     TATTTCCTGC AGCGTTTCGA GCCTTGCCAA CACAGGAGCT TGATCAAGCC AAGTGGG
Consensus       TaTTTacTaC agcGcgTaaA ccCTcgcCAA cACaGGa.CT TGAaCA.... ..GaGaG


PKS14           GCG.....TA AAC....TCT CTTCAAGACGGGCC....TT GAACAGAGAG C......
XM_008604818.1  ACGGGTGTTT TGCCCCGTCT CTTCGAGGTTGACCATGTTT GCGCATAATA AGTACTT
Consensus       aCG.....Taa aC....TCT CTTCaAGacgGaCC....TT GaaCAgAaaa a......


PKS14           GATTCG.CCT AC..CGTC.G AGTGC..CAA CGGCAAAAGA CTCGGTAC.. ..CACGC
bassianolide    AGTACATACT GCAGCGTCTG CGTGCGGCGT CTGCGAGAGC GTAATATCTT CACATGC
Consensus       aaTaCa.aCT aC..CGTC.G aGTGC..Caa CgGCaAaAGa cTaagaaC.. ..CAcGC
```

B

```
PKS15           TTGCATA TCATCATCAT TCATTCATCC ATTCATTCAT TCATACATA. CATACATAAT
Beauvericin     TCGCAGA CCAA.TTCTT TCTCTTGGCT GCACATTCCT TCTTACCTCG CAATCATCAC
Consensus       TcGCAgA cCAa.aTCaT TCacTcagCc acaCATTCaT TCaTACaTa. CAaaCATaAc


PKS15           T TGCATATCAT .CATCATTCA TTCATCCATT CATTCAT.TC ATACATACAT ACATA
XM_008604825    T TCCACATCCC GTAACGCTCA TACATTCGTT ATATCAAGCC ATTCGCTGCT TCGCT
Consensus       T TcCAcATCac .cAaCacTCA TaCATcCaTT aaaTCAa.cC ATaCacacaT aCaca


PKS15           CATATCATCA TCATTCATTC ATCCATTCAT TCATTCATAC ATACATACAT AATTAGA
Bassianolide    AAGAA.TTCA TCAATC.TAC ATC.AGTCGT .CTTTGCTTC ACGTCTCCTC CAGCCAT
Consensus       aAgAa.aTCA TCAaTC.TaC ATC.AgTCaT .CaTTcaTaC AcacaTaCac aAgcaaa


PKS15           TGCATATCAT CATCATTCAT TCATCCATTC ATTCATTCAT ACATACATAC ATAATTA
XM_008597724.1  ACACTATTTT CACCTTGC.T TCTTCACTTC ACTTCTTCCT CTTGACA... GTACCCA
Consensus       acaaTATcaT CAcCaTgC.T TCaTCaaTTC AcTcaTTCaT acagACA... aTAaccA
```

Figure S13. Similarities between the promoters of genes (A) *pks14* or (B) *pks15* with those of genes in the beauvericin and bassianolide biosynthetic clusters.
